# A systematic review on wearable-enabled remote health monitoring

**DOI:** 10.1177/20552076261428387

**Published:** 2026-02-27

**Authors:** Rita Ribeiro, Rafael Martins, Hugo Pereira, Vítor Crista, Júlio Souza, Rute Almeida, Diogo Martinho, Luís Conceição, Alberto Freitas, Goreti Marreiros

**Affiliations:** 1 70922 GECAD – Research Group on Intelligent Engineering and Computing for Advanced Innovation and Development, ISEP, Polytechnic of Porto, Porto, Portugal; 2 RISE-Health, Department of Community Medicine, Information and Health, Decision Sciences, Faculty of Medicine, 26705 University of Porto, Porto, Portugal

**Keywords:** Wearable technologies, remote health monitoring, artificial intelligence, patient engagement, health data analytics

## Abstract

**Objective:**

This systematic review investigates the application of wearable technologies for remote health monitoring, with a particular focus on their effectiveness in improving patient care and the current state of technological integration.

**Methods:**

Following the Preferred Reporting Items for Systematic Reviews and Meta-Analyses methodology, a comprehensive search was conducted for studies published between 2020 and 2025. A total of 55 studies were selected, and data were extracted regarding study design, wearable types, feedback mechanisms, and analytical approaches.

**Results:**

The analysis reveals a significant reliance on traditional statistical methods, with limited integration of advanced artificial intelligence techniques despite their potential to improve predictive capabilities. The review emphasizes the importance of personalized feedback mechanisms in promoting patient engagement and adherence. Furthermore, a notable gap was identified in research addressing cognitive and psychological well-being compared to physical health monitoring.

**Conclusion:**

The findings highlight the need for more rigorous methodologies, including scientific clinical trials, to strengthen the evidence base. Future work should prioritize the integration of machine learning and adopt a more holistic approach to provide a comprehensive understanding of the state-of-the-art and inform future research directions.

## Introduction

With the different technological advancements, remote health monitoring (RHM) systems have emerged as an essential tool to track the health and well-being of general population.^1^ Wearable devices play a crucial role in RHM^2,3^ by enabling continuous, real-time collection of physiological data, such as heart rate and respiratory rate, directly from the user. These devices offer a non-invasive, convenient solution for managing chronic conditions like diabetes,^4^ cardiovascular^5–7^ and respiratory^8–10^ diseases, providing more detailed insights and enhancing the personalization of care. RHM technology has the potential to offer personalized care and support, addressing the specific needs of each individual while promoting active and healthy lifestyles. The concept of remote patient monitoring involves the use of digital devices to collect and process health-related data from patients, which can then be utilized by healthcare professionals or caregivers. RHM systems have gained attention for their ability to provide real-time insights into patients’ health, allowing for timely interventions and reducing the need for frequent in-person visits. This approach is particularly beneficial for individuals with chronic conditions, as it helps monitor their health remotely, reduces hospital readmissions, and ensures continuous care. In this context, wearable devices such as fitness trackers, smartwatches, and specialized medical devices are used to capture objective data, such as step count, heart rate, blood pressure, and sleep patterns.^11–15^ These devices are often paired with applications, usually mobile or web-based, that allow users to track their health metrics, provide personalized feedback, and facilitate the collection of additional self-reported information that allow users to track their health metrics and receive personalized feedback.^16,17^

However, the sheer volume and velocity of physiological data generated by continuous monitoring present a significant processing challenge. In this context, artificial intelligence (AI) and machine learning (ML) have emerged as critical components for transforming these raw sensor outputs into actionable clinical insights. By enabling automated anomaly detection, predictive analytics, and personalized feedback loops, AI applications hold the promise of shifting remote monitoring from a passive data collection exercise to a proactive tool for early intervention and precision medicine.

This work reviews several studies focusing on the application, intervention strategies, and evaluation methods for RHM technologies. It explores various study designs, including the duration of trials, the inclusion of users and health professionals, and the types of wearable devices used. The variety of wearable technologies is a key aspect, as different devices offer different capabilities, and their effectiveness depends on how well they align with the needs of each user. Furthermore, the present study discusses the feedback mechanisms incorporated into these systems, such as notifications, alerts, and personalized recommendations, all aimed at improving patient engagement and health outcomes. In addition to objective data collected by wearable devices, self-reported data may play a complementary role in providing context to the information gathered by sensors. While self-reports are not always seen as reliable, due to their subjective nature, they may help to explain factors such as why a medication was missed, or whether a scheduled physical activity was not completed. The use of validated Patient-Reported Outcome Measures may allow for a more comprehensive understanding of a patient's health, incorporating their perspective, opinions, and preferences. The challenges of integrating remote monitoring technologies into healthcare systems are also discussed in this study. These include topics related to device usability, patient compliance, data privacy, and the need for integration with existing healthcare infrastructures.

Despite these challenges, the potential benefits of RHM systems, such as improved health outcomes, increased patient autonomy, and reduced healthcare costs, make them a promising avenue for advancing healthcare delivery.

While previous systematic reviews have addressed general aspects of remote monitoring,^18–20^ or specific outcomes such as safety and costs,^21^ many do not fully capture the recent explosion of consumer-grade wearable sensors. Furthermore, while some recent reviews focus on the healthcare practitioners’ perspective,^22^ there is a need to synthesize the technological evolution specifically regarding feedback systems and AI integration.

Consequently, literature prior to 2020 often addresses technologies or adoption barriers that are no longer representative of the current state-of-the-art.^19,20^

This review addresses this gap by synthesizing recent evidence (2020–2025) with a specific focus on the effectiveness of continuous monitoring via wearables. Unlike earlier or broader syntheses, this work specifically examines the integration of AI and feedback mechanisms. Finally, the study highlights the need for further research to optimize wearable devices for remote monitoring, particularly within the context of personalized care.

## Methodology

Conducting a systematic review requires a robust and standardized methodology to ensure transparency, reproducibility, and the quality of the results obtained. In this context, the Preferred Reporting Items for Systematic Reviews and Meta-Analyses (PRISMA) framework was adopted, widely recognized as an essential guideline for systematic reviews.^23^ The review was also registered in the International Prospective Register of Systematic Reviews (PROSPERO) under the number CRD420251156256. The PROSPERO protocol is available in the supplementary material.

The application of the PRISMA methodology structured the process into well-defined stages, from formulating the research question to the final inclusion of studies. These stages included identifying relevant data sources, defining inclusion and exclusion criteria, conducting the selection and data extraction process, and synthesizing the results. Throughout each phase, flowcharts and support tables were utilized to ensure clarity in documenting and reporting the applied criteria and decisions. The PRISMA checklist is available in the supplementary material. The following sections detail the main steps and tools employed in executing this process.

### Research questions

The growing adoption of wearable technologies is transforming the way health is monitored and managed remotely, providing more continuous and personalized communication between patients and healthcare professionals. With the ability to collect physiological data in real time, these devices offer new possibilities for preventive health interventions and more informed treatments, expanding the reach of medical care beyond the physical boundaries of clinics and hospitals. However, the variety of devices, technologies, and methods employed creates a diverse and constantly evolving field of research, which requires a detailed review to identify the state-of-the-art in the use of these technologies for RHM.

Thus, the main question defined for this systematic review was: RQ1—“What is the current state-of-the-art regarding the use of wearable technologies for remote health monitoring?”

To answer this question, four secondary research questions were formulated, as presented in Table 1, which focus this state-of-the-art exploration onto the types of wearable technologies available, the medical areas most impacted, the feedback strategies employed, and the most common AI methods in the context of remote monitoring.

**Table 1.** Secondary research questions defined.

Identifier	Research question
SRQ1	What are the main wearables technologies employed in the field of remote health monitoring?
SRQ2	What are the health domains most covered by currently available wearable technologies?
SRQ3	What are the most frequent feedback strategies/mechanisms considered in the context of remote health monitoring?
SRQ4	Which artificial intelligence methods are most used in the context of remote health monitoring?

The first secondary research question, “What are the main wearable technologies employed in the field of remote monitoring?”, plays a crucial role in mapping the current technological ecosystem. By identifying the most common devices and systems, it becomes possible to understand the capabilities, limitations, and availability of these technologies, providing a solid foundation for assessing the state-of-the-art.

The subsequent question, “What are the health domains most covered by currently available wearable technologies?”, explores the fields of medicine where these technologies have the greatest relevance. This analysis is crucial to identify sectors where wearables have already demonstrated significant impact, such as cardiology, neurology, or geriatrics, while also highlighting opportunities for application in less-explored areas.

The third research question, “What are the most frequent feedback strategies/mechanisms considered in the context of remote monitoring?”, investigates how remote monitoring systems can communicate information and responses to health-related events, such as alerts or advice, to both users and healthcare professionals. This aspect is essential for understanding the effectiveness of feedback systems, whether through real-time notifications, detailed reports, or alerts, and their impact on patient adherence to treatment and medical responsiveness.

Finally, the question: “Which artificial intelligence methods are most used in the context of remote monitoring?”, examines the role of AI in enhancing the capabilities of wearable devices. Methods such as ML or deep learning are frequently used for data analysis, pattern recognition, and clinical predictions. This analysis allows for an evaluation of how wearable data is processed to improve clinical outcomes and decision-making.

### Definition of search strategy

To conduct a comprehensive and methodologically rigorous systematic review of the use of wearable technologies in RHM, a structured search process, definition of terms, and study selection criteria were established. This process aims to ensure that this review captures the state-of-the-art in the field and includes only studies of high relevance and scientific quality.

First, the Definition of Search Strategy section describes the approach used to select reliable and relevant databases, ensuring broad and adequate coverage of scientific literature. Next, the Definition of Search Terms section presents the specific search terms that were defined to capture the main concepts and variations related to the topic, such as wearable technologies, remote monitoring, and home contexts. These terms were strategically combined to maximize the relevance of the retrieved results.

Finally, the Study Selection and Data Collection Process sections detail the inclusion and exclusion criteria applied to filter the studies and ensure the relevance of the extracted data. This process aims to select studies that directly examine the application of wearable sensors in remote patient monitoring, focusing on recent research and results evaluated with real users.

This set of steps allows for a rigorous and targeted selection of studies, facilitating an in-depth and accurate analysis of the state-of-the-art in wearable technologies applied to remote health.

#### Information sources

With this review encompassing both the fields of medicine and technology, four electronic databases, presented in Table 2, were searched to ensure adequate coverage of the most significant studies and publications in RHM. Each database was chosen for its relevance to the field and its potential to provide high-quality, up-to-date studies, with PubMed as the main source for studies in the field of medicine, and the remaining three databases, Web of Science, IEEE Xplore, and Science Direct, covering the technological aspect of this systematic review's research question. In this context, Science Direct was preferred over Scopus to reduce redundancy with the other broad-scope databases, while still ensuring access to high-impact full-text publications from Elsevier.

**Table 2.** Information sources searched.

Identifier	Database	URL
ED1	PubMed	https://www.ncbi.nlm.nih.gov/pubmed/
ED2	Web of Science	https://login.webofknowledge.com/
ED3	IEEE Xplore	https://ieeexplore.ieee.org/Xplore/home.jsp
ED4	Science Direct	https://www.sciencedirect.com/

In addition to the database search, reference tracking of all included articles and relevant reviews was performed to identify further studies that met the eligibility criteria but were not captured through the primary search strategy.

#### Definition of search terms

To ensure the identification of the most relevant articles related to the use of wearable technologies in RHM, specific search terms were defined that cover the main aspects of this topic. These terms were chosen to encompass different variations and synonyms used in the literature and were combined into search strings to optimize the retrieval of relevant results. The scope defines the thematic boundaries of the search, and the categories chosen include *Remote Healthcare*, *Domiciliary Context*, and *Wearables*. Table 3 presents the string terms used for each category.

**Table 3.** Search strings utilized.

Scope category	String
Remote healthcare	(“Remote Monitoring” OR “Digital Health” OR “eHealth” OR “mHealth” OR “mobile health” OR “Telehealth”) **AND**
Domiciliary context	(“home” OR “domestic” OR “domiciliary”) **AND**
Wearables	(“wearable” OR “wearables”)

These search strings were combined into a single query and were employed in all previously defined databases, allowing the capture of relevant articles that address the intersection between remote monitoring, home context, and wearable device use. The search was restricted to the Title and Abstract fields and extended to Author Keywords when supported by the database.

#### Eligibility criteria

To ensure the relevance and quality of the studies included in this systematic review, strict inclusion and exclusion criteria were defined. These criteria aim to filter studies that directly address the use of wearable sensors in the context of RHM in domiciliary healthcare settings, as well as to exclude sources that do not meet the scope of the review. Table 4 presents the defined inclusion criteria, which were applied such that a study had to meet all criteria to be included in this review. Table 5, on the other hand, presents the defined exclusion criteria, where a study was excluded if it met at least one of them.

**Table 4.** Inclusion criteria defined.

Identifier	Inclusion criteria
IC1	The source explores how wearable sensors can be integrated into domiciliary healthcare settings
IC2	The source reports tests and results performed with real users
IC3	The study includes detailed analytics applied to the collected data in the context of patient remote monitoring.

**Table 5.** Exclusion criteria defined.

Identifier	Exclusion criteria
EC1	Sources not written in English
EC2	Sources published before 2020
EC3	Sources besides original research journal articles, chapters, conference proceedings and books
EC4	Duplicated sources
EC5	Sources that do not present studies related to domiciliary healthcare remote monitoring
EC6	Sources that do not present studies related to the use of wearable sensors to monitor patients remotely

The decision to restrict the search to studies published from 2020 onwards was driven by the significant technological shift from consumer-grade fitness trackers to clinical-grade biometric sensors observed in recent years.^2^ Furthermore, the COVID-19 pandemic catalyzed a paradigm shift in the adoption of remote patient monitoring, transforming it from an experimental approach into a standard component of domiciliary care.^24^ Consequently, literature prior to this period often addresses technologies or adoption barriers that are no longer representative of the current state-of-the-art.

Regarding source types, this review intentionally prioritized peer-reviewed literature, including journal articles and conference proceedings. To ensure the scientific validity and reliability of the clinical and technical outcomes reported, preprints and clinical trial registries were excluded, as they contain preliminary data that have not yet undergone the rigorous independent scrutiny required for evidence-based health recommendations.

### Identification and screening

Based on the composed search query and by reference tracking, a total of 757 studies were identified, last consulted on 3 October 2025. Prior to screening, 241 duplicate records were removed using the platform Rayyan (https://www.rayyan.ai), that has a semi-automatic tool for this step.

Due to the elevated volume of studies retrieved, seven researchers were enlisted to contribute to the screening phase (RR, HP, RM, VC, JS, DM, LC). For each of the 516 unique articles, two independent and randomly assigned reviewers screened its title and abstract for eligibility based on the previously defined inclusion and exclusion criteria. The screening was conducted using the Rayyan platform in “blind mode” to minimize bias, ensuring that reviewers assessed records independently. In cases of conflicting decisions, the third reviewer served as the final adjudicator to ensure consistent application of the eligibility criteria. With 384 studies being excluded as a result of this screening phase, 132 articles were marked as sought for full-text retrieval.

### Eligibility and quality assessment

From the 132 studies sought for retrieval, the full text of five studies could not be obtained.

Five reviewers (RR, HP, RM, VC, JS) evaluated the full-text content of the remaining studies (n = 127) according to the defined inclusion and exclusion criteria, with any disagreement being resolved by a third reviewer. This step resulted in the exclusion of 63 studies.

Before being included in this review, the remaining 64 studies underwent a quality assessment, where they were excluded if the study's duration was not at least 1 week, or if the study did not have at least 10 participants. This resulted in the exclusion of an additional nine articles, with a final total of 55 studies being selected to be included for review, analysis, and information extraction.

**Figure 1.**
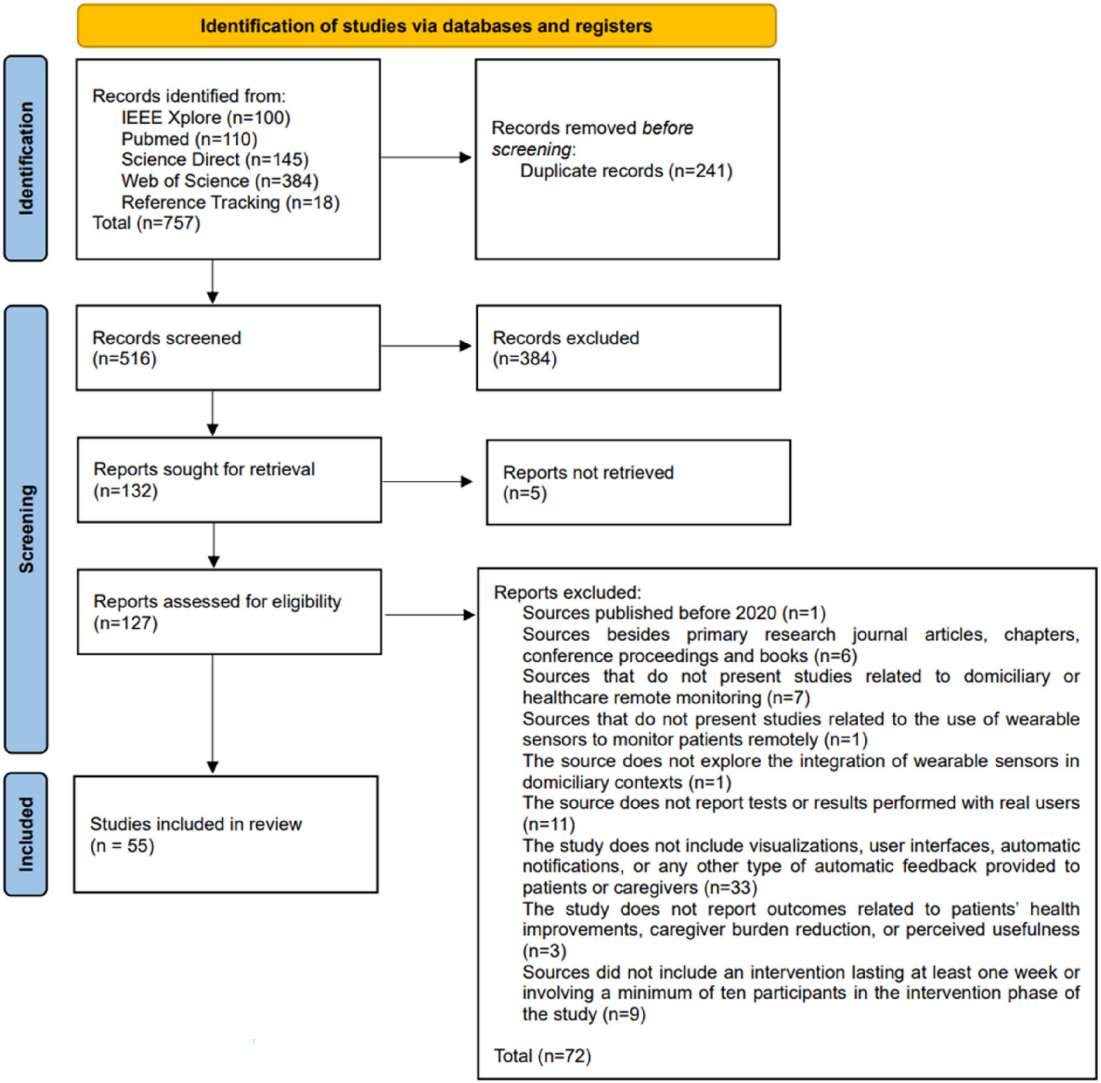
PRISMA flow diagram. PRISMA: Preferred Reporting Items for Systematic Reviews and Meta-Analyses.

The full review process, conducted in accordance with the PRISMA methodology, is illustrated in Figure 1, and the full list of included studies is available in the Supplementary Material.

### Risk of bias assessment

To ensure a rigorous evaluation of the methodological quality of the included studies, an assessment of the risk of bias was conducted using validated and widely recognized tools.^25,26^ Given the heterogeneity of study designs identified in this review, the selection of an appropriate tool for each type of study required careful consideration. Only studies that included a comparator group were eligible for formal risk-of-bias assessment, in alignment with standard recommendations for both tools used.

Accordingly, two instruments were employed. For randomized controlled trials (RCTs), the Risk of Bias 2 (RoB 2) tool was applied,^27^ which evaluates risks related to the randomization process, deviations from the intended interventions, missing outcome data, outcome measurement, and selection of the reported results. For non-randomized intervention studies with comparator groups, the Risk of Bias In Non-randomized Studies of Interventions (ROBINS-I) tool was used.^28^ This tool assesses bias across multiple domains, including confounding, selection of participants, classification of interventions, deviations from intended interventions, missing data, measurement of outcomes, and selection of the reported results.

The RoB 2 tool classifies studies into three levels of risk: low risk, some concerns, and high risk. The ROBINS-I tool classifies studies into four levels: low, moderate, serious, and critical risk of bias. For both instruments, an overall judgment was derived from the domain-level assessments, with particular emphasis placed on the primary outcome of each study.

All assessments were performed independently by two reviewers. Discrepancies were discussed and resolved through consensus, ensuring methodological rigor and minimizing subjectivity in the evaluation process.

### Data collection process

To organize the extraction of information from the studies included in this systematic review, a supplementary table (provided as a Microsoft Excel spreadsheet) was created and used by the reviewers (RR, HP, RM, VC, JS) to collect the following data fields: author name and year, app name or study name, country, study design, population characteristics (number of participants, age, and setting), duration of the study intervention, intervention characteristics, monitoring characteristics, comparator/control group, study target / orientation of the system (for the patient or health professional), categorization (transitional care, rehabilitation, prevention, monitoring, and diagnosis), type of wearables, data collection, data visualization, feedback system, ML models, and main findings (for the patient or health professional). In cases where the information was not present or unclear, it was flagged in our Microsoft Excel spreadsheet as Not Applicable (NA) or described as not having been specified.

### Outcomes

The primary outcomes of interest in this review were related to the application and effectiveness of wearable-based remote monitoring systems in domiciliary context. Specifically, outcomes were grouped according to the four research questions and included:

- wearable technologies employed (type, placement, origin, sensing modality),
- remote monitoring focus and clinical domain,
- feedback strategies and mechanisms used to support patient or clinician decision-making, and
- analytical and AI methods applied to the collected data.

All results reported in the included studies that were compatible with these outcome domains were extracted, regardless of measurement scale, time point, or analytical approach. No restrictions were applied regarding which findings within each study were eligible for extraction.

## Results

This section describes and discusses the results retrieved from the 55 research works included in this review regarding different examined categories. Afterwards, it depicts how each research question was answered according to the information obtained in the studies.

### Region/area of study

The studies exhibit global distribution, but the evidence base is dominated by high-income countries. The United States accounts for the largest share, followed by a smaller cluster of European and Asian contributions. Low- and middle-income regions are minimally represented, indicating that most evidence on remote monitoring is generated in settings with strong digital infrastructure. This geographic imbalance limits the generalizability of findings to underrepresented healthcare systems. Table 6 summarizes the number of studies per region and lists the specific papers included in each.

**Table 6.** Region/area intervention of the included studies.

Region/Area	Studies	Number of studies
Australia	Indraratna et al.^24^ Ha et al.^29^	2
Belgium	Leenen et al.^30^	1
Canada	Wu et al.^31^ Hesketh et al.^32^	2
China	Liu et al.^33^ Li et al.^34^	2
France	Cochen De Cock et al.^35^	1
Germany	Oftring et al.^36^ Klein et al.^37^ Wurzer et al.^15^ Gaßner et al.^38^	4
Hong Kong	Toh et al., (2025)^39^ Wong et al.^40^ Toh et al., (2023)^41^	3
Italy	Motolese et al.^42^ Bonometti et al.^43^	2
Japan	Sze et al.^44^ Kikuchi et al.^45^	2
Netherlands	van Ede et al.^46^ Breteler et al.^11^ van den Bergh et al.^47^ Leenen et al.^30^ Khusial et al.^48^	5
Norway	Stubberud et al.^49^	1
South Africa	Davies et al.^50^	1
South Korea	Chae et al.^51^	1
Spain	Sañudo et al.^52^	1
Taiwan	Tzeng et al.^53^ Lin et al.^54^ Li et al.^55^ Chen et al.^56^	4
United Kingdom	Althobiani et al.^57^ Hesketh et al.^32^ Khusial et al.^48^	3
United States	Charkviani et al.^58^ Anand et al.^59^ Loh et al.^60^ O’Connor et al.^61^ Patel et al.^13^ Leitner et al.^12^ Batsis et al.^17^ Wilmink et al.^62^ Bell et al.^63^ Mehta et al.^64^ LeBaron et al.^65^ Walter et al.^66^ Fanning et al.^67^ Moore et al.^68^ Matthews et al.^69^ Adams et al.^16^ Ding et al.^70^ Downing et al.^71^ Harzand et al.^72^ Blair et al.^73^ Purnell et al.^14^ Leitner et al.^74^	22

### Study design

A total of 55 studies were included in this systematic review, encompassing a broad spectrum of methodological designs. The evidence base consisted of RCTs, non-randomized intervention studies, clinical studies, observational studies, feasibility studies, pilot studies, mixed-methods studies, qualitative research, and a small subset of articles with unspecified designs. Several studies were classified into multiple methodological categories due to hybrid or overlapping research features. For example, Blair et al.^73^ was classified as both an RCT and a pilot study; Chen et al.^56^ and Sze et al.^44^ were each categorized as non-randomized intervention and pilot studies; and Wu et al.^31^ simultaneously met criteria for non-randomized intervention, observational, and feasibility study designs. Similarly, Althobiani et al.,^57^ Breteler et al.,^11^ and Davies et al.^50^ were each included within both observational and feasibility categories. In addition, van den Bergh et al.^47^ and the study associated with LeBaron et al.^65^ were classified across three categories (observational, feasibility, and pilot studies), while Duarte-Rojo et al.^75^ and Ha et al.^29^ fell under both feasibility and pilot study classifications. These overlaps underscore the methodological diversity and complexity of literature and reflect the exploratory nature of many investigations. A detailed breakdown of these classifications is presented in Table 7, titled References by Study Design.

**Table 7.** References by study design.

Study design	Studies	Number of studies
Randomized controlled trials (RCTs)	Adams et al., (2021)^16^ Anand et al., (2021)^59^ Bell et al., (2020)^63^ Blair et al., (2021)^73^ Indraratna et al., (2021)^24^ Khusial et al., (2020)^48^ Sañudo et al., (2024)^52^ Hesketh et al., (2025)^32^ Mehta et al., (2020)^64^ Li et al., (2025)^34^ Tzeng et al., (2025)^53^ Toh et al., (2025)^39^ Fanning et al., (2020)^67^	13
Non-randomized intervention studies	Batsis et al., (2021)^17^ Chen et al., (2020)^56^ Harzand et al. (2023)^72^ Kikuchi et al., (2021)^45^ Li et al., (2024)^55^ Sze et al., (2023)^44^ Wu et al., (2024)^31^ Leenen et al., (2023)^30^ Kikuchi et al., (2021)^45^ Leitner et al., (2024)^74^	10
Clinical studies	Downing et al., (2023)^71^ Leitner et al., (2022)^12^	2
Observational studies	Althobiani et al., (2023)^57^ Breteler et al., (2020)^11^ Chae et al., (2020)^51^ Cochen De Cock et al., (2021)^35^ Davies et al., (2021)^50^ Patel et al., (2022)^13^ van den Bergh et al., (2023)^47^ Wu et al., (2024)^31^ LeBaron et al., (2023)^65^ Liu et al., (2022)^33^ Walter et al., (2023)^66^	11
Feasibility studies	Althobiani et al., (2023)^57^ Breteler et al., (2020)^11^ Davies et al., (2021)^50^ Ding et al., (2021)^70^ Duarte-Rojo et al., (2023)^75^ Ha et al., (2022)^29^ Motolese et al., (2023)^42^ van den Bergh et al., (2023)^47^ van Ede et al., (2022)^46^ Wu et al., (2024)^31^ Wurzer et al., (2021)^15^ Klein et al., (2024)^37^ LeBaron et al., (2023)^65^ Stubberud et al., (2020)^49^ Moore et al., (2025)^68^	15
Pilot studies	Blair et al., (2021)^73^ Bonometti et al., (2023)^43^ Charkviani et al., (2023)^58^ Chen et al., (2020)^56^ Duarte-Rojo et al., (2023)^75^ Gaßner et al., (2022)^38^ Ha et al., (2022)^29^ Loh et al., (2022)^60^ Sze et al., (2023)^44^ van den Bergh et al., (2023)^47^ Wilmink et al., (2020)^62^ Wong et al., (2022)^40^ O’Connor et al., (2025)^61^ Lin et al., (2024)^54^ LeBaron et al., (2023)^65^	15
Mixed-methods studies	Toh et al., (2023)^41^ Oftring et al., (2025)^36^	2
Qualitative studies	Purnell et al., (2022)^14^	1
Not reported	Matthews et al., (2024)^69^	1

The diversity of study designs illustrates the evolving maturity of research on remote monitoring technologies. While the inclusion of RCTs offers high-quality evidence, the predominance of non-randomized, observational, feasibility, and pilot studies indicates that this field is still in an early developmental phase, with many interventions undergoing preliminary evaluation rather than full-scale effectiveness testing. This imbalance highlights a clear gap in the evidence base. The limited number of rigorously controlled trials constrains the strength of conclusions that can be drawn. Increasing the prevalence of well-designed RCTs would provide more robust estimates of effectiveness, support stronger causal inference, and enhance confidence in the applicability and scalability of remote monitoring technologies in real-world clinical settings.

### Participants characteristics

**Figure 2.**
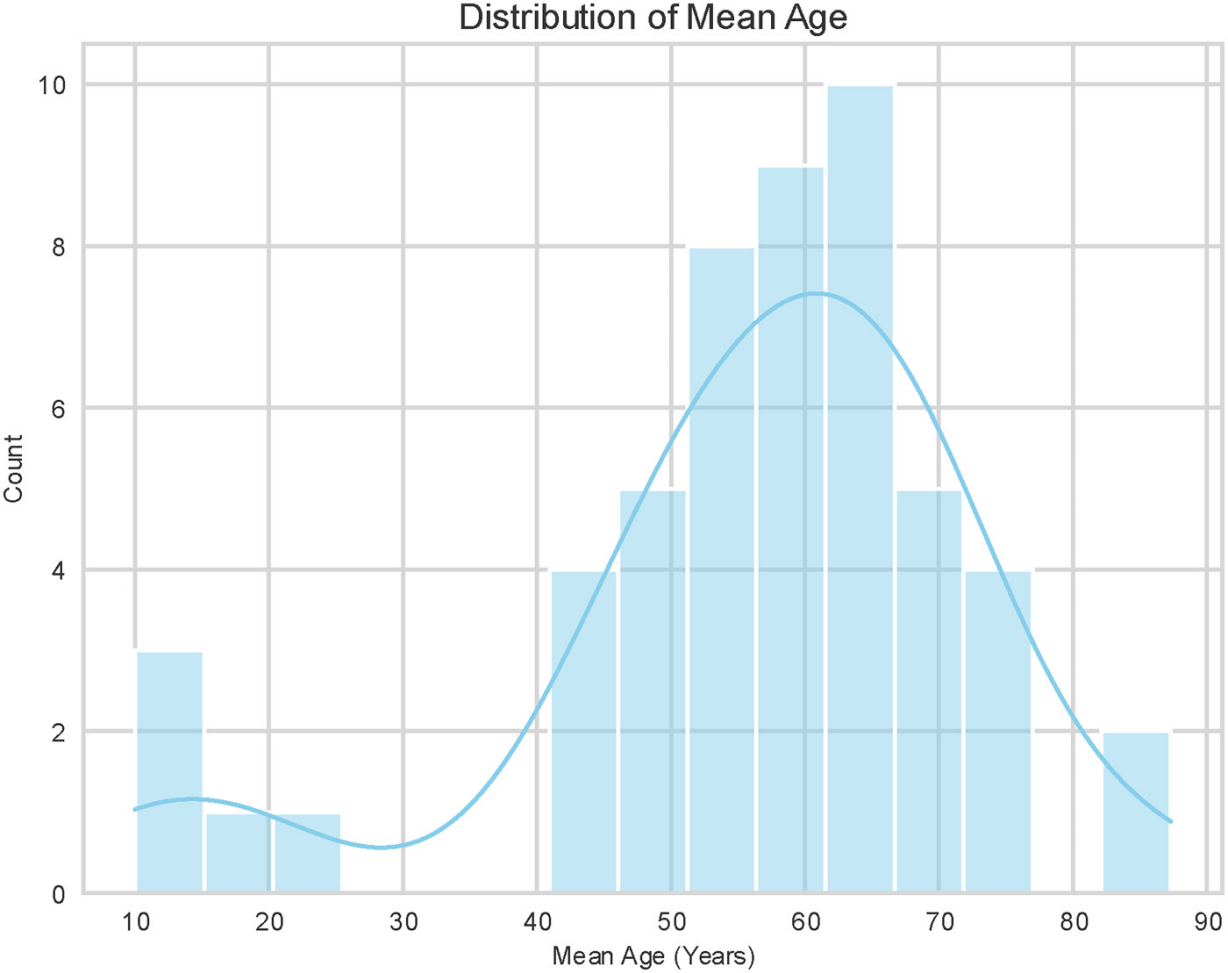
Distribution of mean age.

The distribution of mean participant ages across the included studies is illustrated in Figure 2. The data reveals a bimodal distribution, heavily weighted towards older adult populations. The vast majority of studies focused on participants with a mean age between 50 and 75 years, with the highest frequency of studies (the peak of the distribution) reporting a mean age in the 60–65-year range. This suggests a strong research emphasis on aging populations or conditions prevalent in later life (e.g. COPD, heart failure). On the other hand, a smaller, distinct cluster of studies focused on pediatric and adolescent populations, with mean ages ranging from approximately 10 to 20 years. Notably, there is a relative scarcity of studies targeting young-to-middle-aged adults (ages 25–45) in this review.

**Figure 3.**
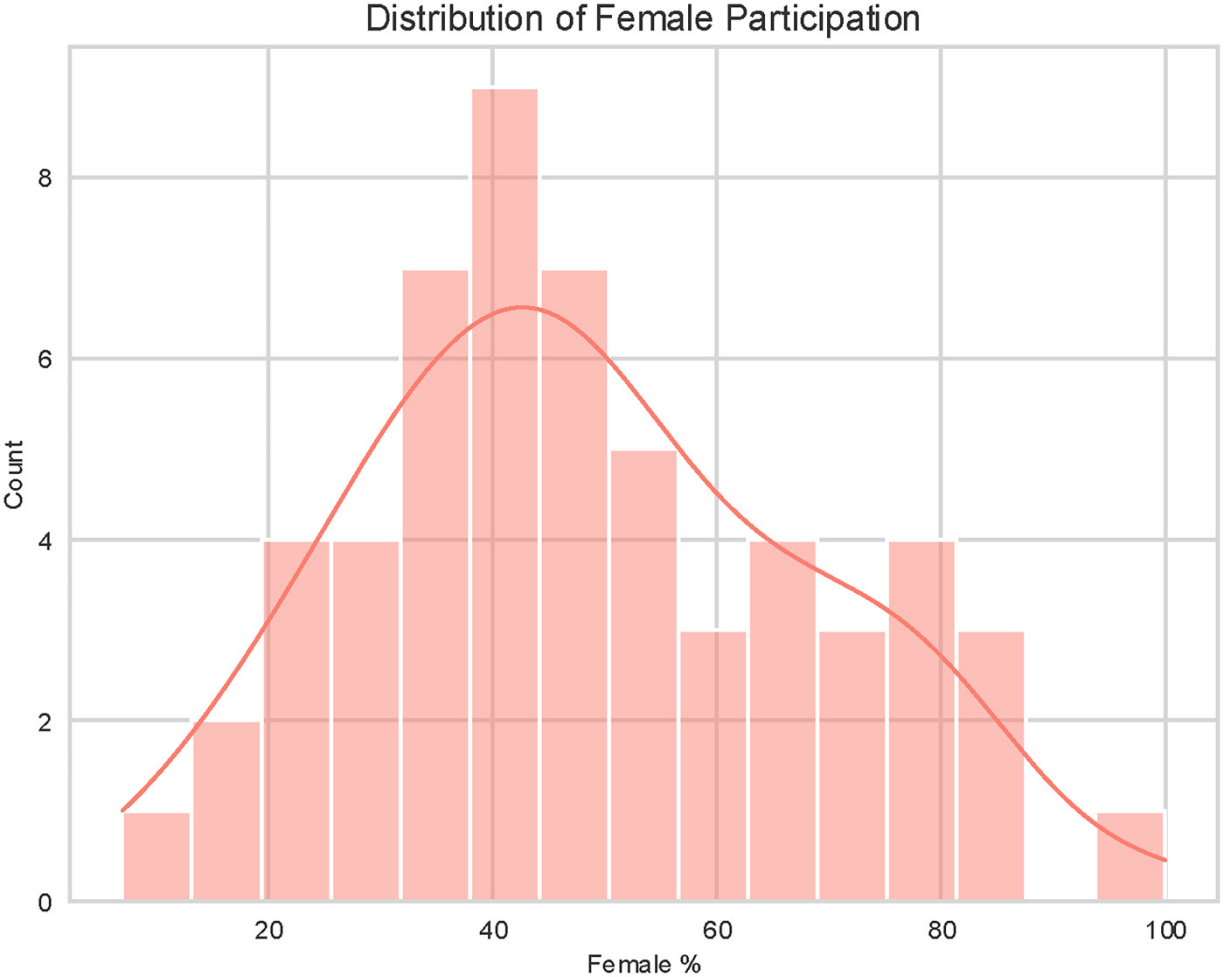
Distribution of female participation.

The gender composition of the included studies is displayed in Figure 3. The distribution of female participation varies widely across the selected literature, ranging from approximately 10% to nearly 100%. The distribution is relatively wide, with a notable peak in the 40–45% range, indicating that a significant portion of studies utilized samples with a slight male majority or a near-balanced gender ratio.

However, the data also reveals considerable heterogeneity. While the central tendency leans towards mixed-gender cohorts, there is a distinct subset of studies with high female representation (>75%), likely reflecting condition-specific research (e.g. autoimmune diseases or specific cancers). In opposition, studies with very low female participation (<20%) are less frequent but present.

**Figure 4.**
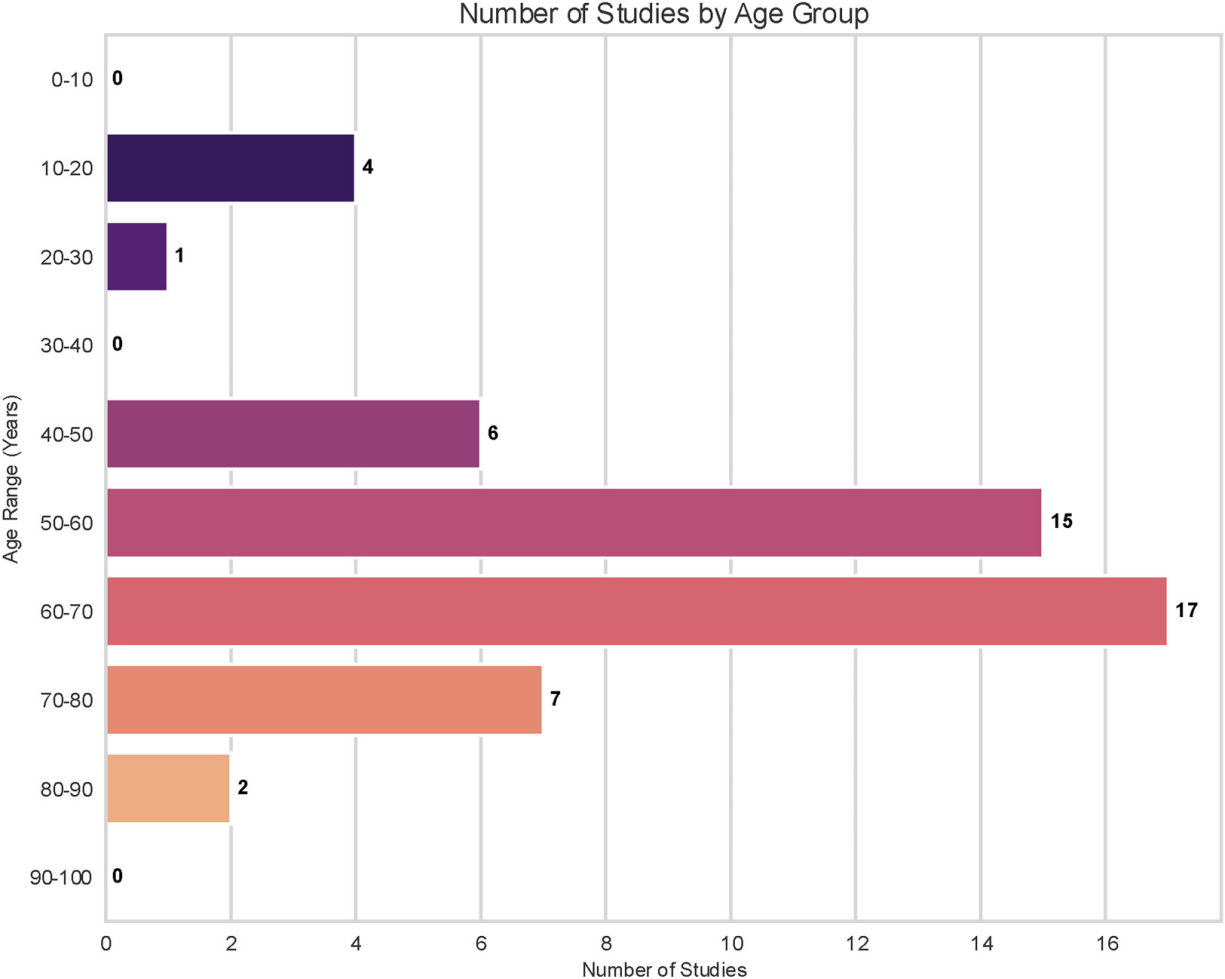
Number of studies by age group.

The distribution of studies across specific age decades is detailed in Figure 4. Consistent with the mean age distribution observed in Figure 2, the majority of research is concentrated on older adults. The highest volume of studies targeted participants in the 60–70-year age range (n = 17), followed closely by the 50–60-year group (n = 15). There is a notable gap in research targeting young-to-middle-aged adults, with only a single study falling into the 20–30-year category and none in the 30–40-year range. A smaller, secondary focus is evident in the adolescent population (10–20 years), which was the subject of four studies.

**Figure 5.**
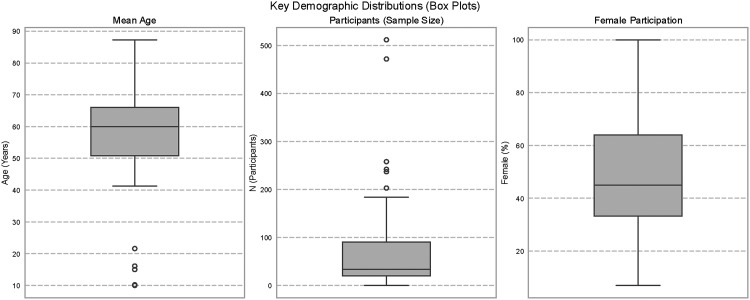
Key demographic distributions.

The box plot analysis in Figure 5 highlights key demographic trends across age, sample size, and gender. The Left Panel displays a median participant age of approximately 60 years, marked by distinct outliers in the pediatric range. Regarding study scale, the Middle Panel illustrates a highly biased distribution with a median sample size of fewer than 50 participants; however, distinct outliers upwards of 500 participants suggest the inclusion of large-scale trials. Finally, the Right Panel reflects the heterogeneity of gender composition, showing an interquartile range of female participation that spans from approximately 35% to 65%.

**Figure 6.**
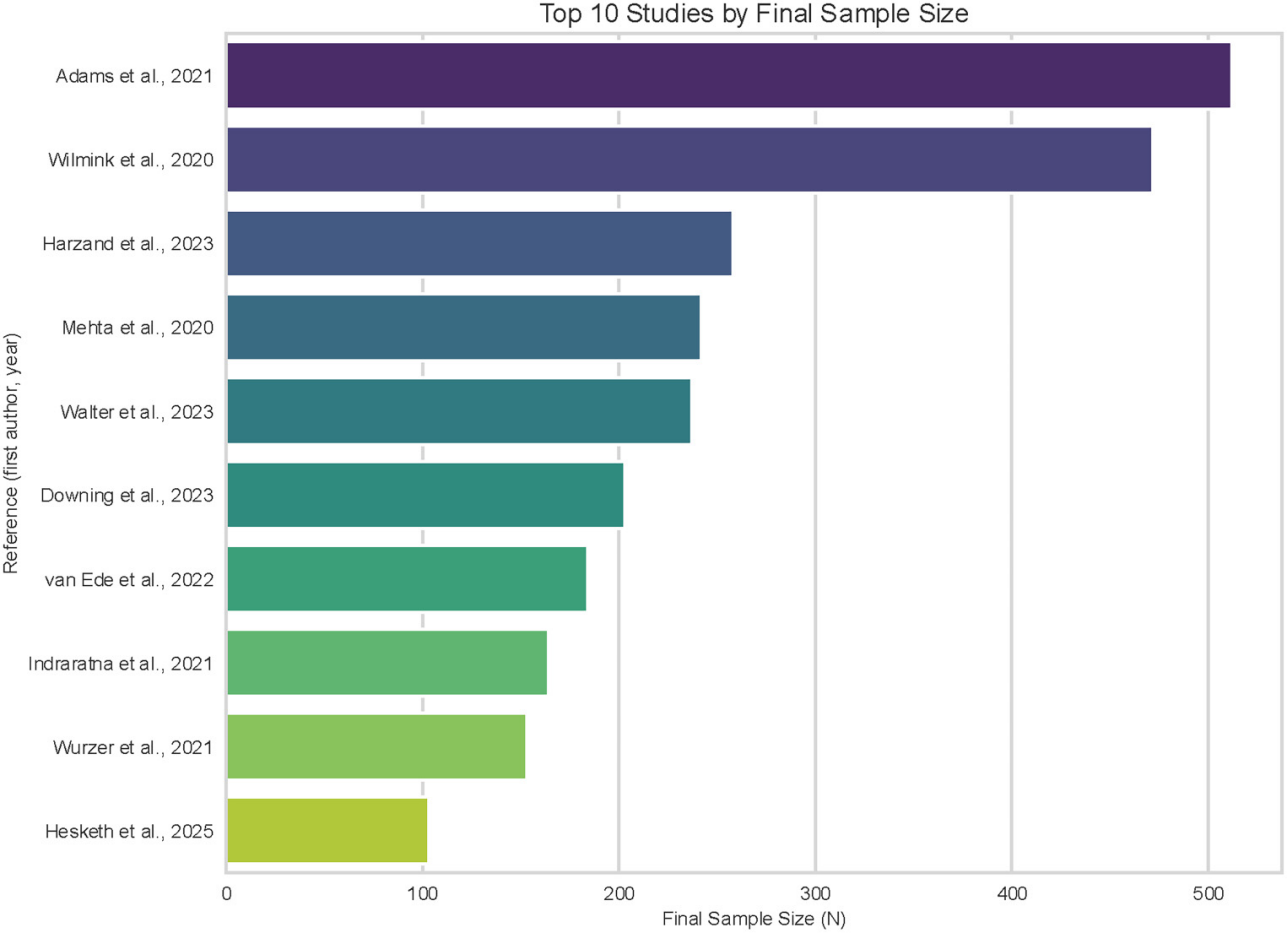
Top 10 studies final sample size.

To further elucidate the distribution of these larger cohorts, Figure 6 details the top 10 studies by final sample size. Adams et al.^16^ and Wilmink et al.^62^ represent the most substantial contributions to the review, with sample sizes approaching or exceeding 500 participants. This visualization highlights a gradation in study magnitude, with the remaining top-tier studies ranging between approximately 100 and 250 participants. These larger datasets primarily stem from trials involving established clinical populations, such as those in cardiac rehabilitation or assisted living communities.

### Duration of study intervention

In terms of duration, as presented in Table 8, 45 of the 55 studies conducted their participant monitoring trials for at least 4 weeks. Of those, 13 studies monitored each patient for at least 24 weeks (approximately 6 months), with the article by Wilmink et al.^62^ reporting a monitoring period of 105 weeks (24 months). The remaining 10 studies reported shorter monitoring durations, ranging from 1 week to 3 weeks.

**Table 8.** References by duration of study.

Duration of study	References
1–3 weeks	Breteler et al., (2020)^11^ LeBaron et al., (2023)^65^ Lin et al., (2024)^54^ Patel et al., (2022)^13^ Purnell et al., (2023)^14^ Stubberud et al., (2020)^49^ Toh et al., (2023)^41^ van Ede et al., (2022)^46^ Walter et al., (2023)^66^ Wurzer et al., (2021)^15^
4–23 weeks	Anand et al., (2021)^59^ Bell et al., (2020)^63^ Blair et al., (2021)^73^ Bonometti et al., (2023)^43^ Chae et al., (2020)^51^ Charkviani et al., (2023)^58^ Chen et al., (2020)^56^ Cochen De Cock et al., (2021)^35^ Ding et al., (2021)^70^ Downing et al., (2023)^71^ Duarte-Rojo et al., (2023)^75^ Fanning et al., (2020)^67^ Gaßner et al., (2022)^38^ Ha et al., (2022)^29^ Harzand et al., (2023)^72^ Kikuchi et al., (2021)^45^ Klein et al., (2024)^37^ Li et al., (2025)^34^ Liu et al., (2022)^33^ Loh et al., (2022)^60^ Matthews et al., (2024)^69^ Mehta et al., (2020)^64^ Moore et al., (2025)^68^ Motolese et al., (2023)^42^ O'Connor et al., (2025)^61^ Oftring et al., (2025)^36^ Sañudo et al., (2024)^52^ Sze et al., (2023)^44^ Toh et al., (2025)^39^ Tzeng et al., (2025)^53^ van den Bergh et al., (2023)^47^ Wong et al., (2022)^40^
>=24 weeks	Adams et al., (2022)^16^ Althobiani et al., (2023)^57^ Batsis et al., (2021)^17^ Davies et al., (2021)^50^ Hesketh et al., (2025)^32^ Indraratna et al., (2021)^24^ Khusial et al., (2020)^48^ Leenen et al., (2023)^30^ Leitner et al., (2022)^12^ Leitner et al., (2024)^74^ Li et al., (2024)^55^ Wilmink et al., (2020)^62^ Wu et al., (2024)^31^

While some studies focused only on short-term monitoring, usually to test the feasibility of a system or solution, the majority monitored the participants for at least 4 weeks (1 month), describing the effects of the presented solution on the patients’ health. However, few studies carry out the remote monitoring trials for longer periods of time (of at least 6 months), leaving a gap in the literature for studying the more prolonged exposure of participants to remote monitoring solutions, and its resulting effects on the patients’ health evolution and/or opinion on the comfort/usability of the wearable devices.

### Monitoring characteristics

Studies in this review utilized either a continuous, a periodic, or a combined approach for health monitoring, as demonstrated in Table 9. Studies that employed continuous monitoring (n = 25) focused on 24/7 tracking of metrics such as heart rate, respiratory rate, or physical activity. Periodic monitoring studies (n = 14), on the other hand, involved checking metrics only at set intervals or only during intervention-related events, such as vital sign monitoring during a daily prescribed home exercise. The remaining 17 studies opted for a combined approach, by passively continuously monitoring health data or with additional real-time recommendation/coaching systems, while at the same time scheduling periodic progress assessments or more intensive data collection sessions.

**Table 9.** Monitoring approaches.

Monitoring approach	References
Continuous only	Adams et al., (2022)^16^ Anand et al., (2021)^59^ Blair et al., (2021)^73^ Bonometti et al., (2023)^43^ Breteler et al., (2020)^11^ Charkviani et al., (2023)^58^ Davies et al., (2021)^50^ Ding et al., (2021)^70^ Indraratna et al., (2021)^24^ Moore et al., (2025)^68^ Kikuchi et al., (2021)^45^ LeBaron et al., (2023)^65^ Leitner et al., (2022)^12^ Liu et al., (2022)^33^ O’Connor et al., (2025)^61^ Patel et al., (2022)^13^ Purnell et al., (2023)^14^ Sañudo et al., (2024)^52^ Stubberud et al., (2020)^49^ van Ede et al., (2022)^46^ Walter et al., (2023)^66^ Wilmink et al., (2020)^62^ Wong et al., (2022)^40^ Wu et al., (2024)^31^ Wurzer et al., (2021)^15^
Periodic only	Bell et al., (2020)^63^ Chen et al., (2020)^56^ Cochen De Cock et al., (2021)^35^ Gaßner et al., (2022)^38^ Ha et al., (2022)^29^ Klein et al., (2024)^37^ Leenen et al., (2023)^30^ Leitner et al., (2024)^74^ Lin et al., (2024)^54^ Matthews et al., (2024)^69^ Mehta et al., (2020)^64^ Toh et al., (2023)^41^ Toh et al., (2025)^39^ Tzeng et al., (2025)^53^
Combined (continuous + periodic)	Althobiani et al., (2023)^57^ Batsis et al., (2021)^17^ Chae et al., (2020)^51^ Downing et al., (2023)^71^ Duarte-Rojo et al., (2023)^75^ Fanning et al., (2020)^67^ Harzand et al., (2023)^72^ Hesketh et al., (2025)^32^ Khusial et al., (2020)^48^ Li et al., (2024)^55^ Li et al., (2025)^34^ Loh et al., (2022)^60^ Motolese et al., (2023)^42^ Sze et al., (2023)^44^ van den Bergh et al., (2023)^47^

In terms of evaluation, as presented in Table 10, 15 articles assessed their results by evaluating objective measures obtained only, such as the evolution of the patients’ health before and after the intervention through vital signs or physical performance improvement, exacerbation, or hospital readmission reduction, engagement time with the wearables/apps, or integrity of the data. A total of 34 studies complemented these objective measures with subjective metrics, namely in the form of surveys or questionnaires, to collect insights on the patient's self-perceived physical or mental well-being, or their satisfaction with the health monitoring procedure. The remaining seven studies evaluated their results utilizing solely user-reported questionnaires (answered by patients or health professionals), primarily to assess the systems developed in terms of usability, performance, and user satisfaction.

**Table 10.** Evaluation metrics.

Evaluation metrics	References
Objective only	Adams et al., (2022)^16^ Chae et al., (2020)^51^ Charkviani et al., (2023)^58^ Downing et al., (2023)^71^ Duarte-Rojo et al., (2023)^75^ Kikuchi et al., (2021)^45^ Lin et al., (2024)^54^ Liu et al., (2022)^33^ Leitner et al., (2022)^12^ Matthews et al., (2024)^69^ Patel et al., (2022)^13^ van Ede et al., (2022)^46^ Walter et al., (2023)^66^ Wilmink et al., (2020)^62^ Wong et al., (2022)^40^
Subjective only	Ding et al., (2021)^70^ LeBaron et al., (2023)^65^ Moore et al., (2025)^68^ Purnell et al., (2023)^14^ Stubberud et al., (2020)^49^ Toh et al., (2023)^41^ van den Bergh et al., (2023)^47^
Combined (objective + subjective)	Althobiani et al., (2023)^57^ Anand et al., (2021)^59^ Batsis et al., (2021)^17^ Bell et al., (2020)^63^ Blair et al., (2021)^73^ Bonometti et al., (2023)^43^ Breteler et al., (2020)^11^ Chen et al., (2020)^56^ Cochen De Cock et al., (2021)^35^ Davies et al., (2021)^50^ Fanning et al., (2020)^67^ Gaßner et al., (2022)^38^ Ha et al., (2022)^29^ Harzand et al., (2023)^72^ Hesketh et al., (2025)^32^ Indraratna et al., (2021)^24^ Khusial et al., (2020)^48^ Klein et al., (2024)^37^ Leenen et al., (2023)^30^ Leitner et al., (2024)^74^ Li et al., (2024)^55^ Li et al., (2025)^34^ Loh et al., (2022)^60^ Mehta et al., (2020)^64^ Motolese et al., (2023)^42^ O’Connor et al., (2025)^61^ Oftring et al., (2025)^36^ Sañudo et al., (2024)^52^ Sze et al., (2023)^44^ Toh et al., (2025)^39^ Tzeng et al., (2025)^53^ Wu et al., (2024)^31^ Wurzer et al., (2021)^15^

The diversity in monitoring and evaluation strategies across studies shows the adaptability of wearable and remote monitoring technologies. Both continuous and periodic monitoring were used across the studies, with each approach offering different advantages depending on the context. Continuous monitoring can support timely responses to changes in a patient's condition, which seems particularly useful in more acute or unstable cases. Periodic monitoring appeared more frequently in studies targeting chronic conditions or rehabilitation, possibly reflecting lower demands for real-time tracking in those specific interventions. Still, the choice between these approaches really depends on the specific health condition and the goals of the intervention, and the studies reviewed varied quite a bit in how they measured and reported outcomes. Similarly, objective metrics and user-reported questionnaires were commonly used as strategies to assess the intervention. This mix of methods provides a flexible framework for tailoring remote healthcare interventions to patient needs and resource availability.

### Comparator/control group strategy

A total of 18 studies reported a comparator or control group for intervention assessment, of which seven were RTCs.

In 5 of the 11 studies that reported a comparator group the participants in the comparator group received standard care, with no additional intervention provided.^31,45,55,63,73^ The remaining six studies described a variety of alternative control conditions: Chen et al.^56^ compared a home-based exercise group without wearables, consisting of seven participants, to a motion sensor-assisted rehabilitation group of eight participants, also including 10 additional participants to assess the reliability of the inertial sensor. Another study^44^ established a control group that received routine monitoring without gamified feedback and another^12^ included participants who did not receive any recommendations while being provided with devices for data collection. Chae et al.^51^ described a control group that only received initial instructions on how to perform the exercises, along with a printed handout as a reminder. Additionally, Anand et al.^59^ focused on a control group of participants using wearable activity trackers and receiving fitness professional counseling, while Adams et al.^16^ employed static goals with delayed reinforcement.

While comparator groups were consistently included in the RCTs—as expected by definition—, their use was less common in studies with other designs. Among those that did include a comparator, the variability in methods and intervention types makes it difficult to draw consistent conclusions. This lack of standardization across non-RCTs highlights a gap in the literature and limits the strength of the evidence regarding the relative effectiveness of the interventions studied.

### Study target / orientation

The systems and applications developed in the 55 reviewed studies were categorized according to their target users, explicitly shown in Table 11, as either being oriented towards the patient, towards health professionals, or towards both types of users.

**Table 11.** Study target.

Study target	Studies	Number of studies
Patients + health professionals	Althobiani et al., (2023)^57^ Anand et al., (2021)^59^ Batsis et al., (2021)^17^ Bell et al., (2020)^63^ Bonometti et al., (2023)^43^ Breteler et al., (2020)^11^ Chae et al., (2020)^51^ Chen et al., (2020)^56^ Cochen De Cock et al., (2021)^35^ Davies et al., (2021)^50^ Harzand et al., (2023)^72^ Indraratna et al., (2021)^24^ Kikuchi et al., (2021)^45^ Leenen et al., (2024)^30^ Li et al., (2024)^55^ Li et al., (2025)^34^ Lin et al., (2024)^54^ Loh et al., (2022)^60^ Matthews et al., (2024)^69^ O'Connor et al., (2025)^61^ Purnell et al., (2023)^14^ Toh et al., (2023)^41^ van den Bergh et al., (2023)^47^ Walter et al., (2023)^66^ Klein et al., (2024)^37^ Wong et al., (2022)^40^ Moore et al., (2025)^68^	27
Patients only	Adams et al., (2022)^16^ Blair et al., (2021)^73^ Ding et al., (2021)^70^ Duarte-Rojo et al., (2023)^75^ Fanning et al., (2020)^67^ Gaßner et al., (2022)^38^ Ha et al., (2022)^29^ Hesketh et al., (2025)^32^ Khusial et al., (2020)^48^ LeBaron et al., (2023)^65^ Leitner et al., (2022)^12^ Leitner et al., (2024)^74^ Liu et al., (2022)^33^ Mehta et al., (2020)^64^ Motolese et al., (2023)^42^ Oftring et al., (2025)^36^ Sañudo et al., (2024)^52^ Stubberud et al., (2020)^49^ Sze et al., (2023)^44^ Toh et al., (2025)^39^ Tzeng et al., (2025)^53^ Wu et al., (2024)^31^	22
Health professionals only	Charkviani et al., (2023)^58^ Downing et al., (2023)^71^ Patel et al., (2022)^13^ van Ede et al., (2022)^46^ Wilmink et al., (2020)^62^ Wurzer et al., (2021)^15^	6

The majority of studies (n = 27) were oriented to both patients and health professionals, with the developed systems often encompassing different, but complementary, sets of features to be utilized by each group. The most common model seen in these studies is the use of a patient-dedicated application for data collection, simplified data visualization, and/or feedback, goal or recommendation visualization, while on the other hand providing health professionals with applications or systems that allow for dashboard data monitoring, goal setting, and telecommunication with the patients.

Twenty-two other studies were geared towards patients only, providing them with the aforementioned tools while commonly focusing on a self-monitoring or self-training approach, whereas the remaining six studies were oriented towards health professionals only, again providing them with the previously described monitoring tools, while focusing on an invisible data collection approach for the patients.

These findings highlight the feasibility of three different approaches to RHM, and the significance of clearly defining the orientation of studies related to this field. As can be seen from the articles reviewed, approaching a remote monitoring study from different orientation perspectives seems to heavily influence the systems utilized and their respective necessary functionalities, implying that if the study targets are not clearly defined early on, the systems developed and therefore the results obtained may be significantly negatively affected.

Studies and systems involving both patient and health professionals are shown to be the most sought after, with patient-oriented studies also attracting considerable interest, while studies oriented solely towards health professionals are less explored. This trend may reflect a recognition of the decisive role of patient awareness and active involvement in health monitoring, even in remote and digital settings, as opposed to remote monitoring approaches that aim for data collection and monitoring in a process mostly invisible to the patient. The interest in patient-oriented studies may further reflect this tendency for patient active involvement in the health monitoring process, in this case assigning the primary self-monitoring role to the patients themselves.

### Categorization

To better understand the scope and objectives of the included studies, they were categorized into five main groups, as reported in the summary Table 12, based on their remote monitoring primary focus: Transitional Care, Rehabilitation, Prevention, Monitoring, and Diagnosis.

**Table 12.** Categorization based on remote monitoring primary focus.

Remote monitoring primary focus	Studies	Number of studies
Transitional care	Althobiani et al., (2023)^57^ Bonometti et al., (2023)^43^ Breteler et al., (2020)^11^ Charkviani et al., (2023)^58^ Downing et al., (2023)^71^ Patel et al., (2022)^13^ van Ede et al., (2022)^46^	7
Rehabilitation	Bell et al., (2020) Chae et al., (2020)^51^ Chen et al., (2020)^56^ Cochen De Cock et al., (2021)^35^ Harzand et al., (2023)^72^ Fanning et al., (2020)^67^ Gaßner et al., (2022)^38^ Li et al., (2025)^34^ Ha et al., (2022)^29^ Loh et al., (2022)^60^ O'Connor et al., (2025)^61^ Toh et al., (2023)^41^ Toh et al., (2025)^39^ Tzeng et al., (2025)^53^	14
Prevention	Anand et al., (2021)^59^ Batsis et al., (2021)^17^ Adams et al., (2021)^16^ Blair et al., (2021)^73^ Li et al., (2024)^55^ Duarte-Rojo et al., (2023)^75^ Hesketh et al., (2025)^32^ Leitner et al., (2024)^74^ Moore et al., (2025)^68^	9
Monitoring	Ding et al., (2021)^70^ Davies et al., (2021)^50^ Indraratna et al., (2021)^24^ Kikuchi et al., (2021)^45^ Leenen et al., (2023)^30^ Wilmink et al., (2020)^62^ Lin et al., (2024)^54^ Khusial et al., (2020)^48^ LeBaron et al., (2023)^65^ Matthews et al., (2024)^69^ Wurzer et al., (2021)^15^ Leitner et al., (2022)^12^ Liu et al., (2022)^33^ Mehta et al., (2020)^64^ Motolese et al., (2023)^42^ Oftring et al., (2025)^36^ van den Bergh et al., (2023)^47^ Walter et al., (2023)^66^ Klein et al., (2024)^37^ Sañudo et al., (2024)^52^ Wong et al., (2022)^40^ Stubberud et al., (2020)^49^ Sze et al., (2023)^44^ Wu et al., (2024)^31^	24
Diagnosis	Purnell et al., (2023)^14^	1

Represented by a single study, the categorization in Diagnosis includes research focused on evaluating devices for clinical assessment. This category evaluates the acceptability and usability of a wearable device for sleep health, comparing its performance to traditional diagnostic tools.

Monitoring studies, which constitute the most extensive group in this review, involve tracking health parameters and symptoms over time. Studies in this category focus on the continuous observation of physiological data. This includes the wireless remote home monitoring of vital signs in patients discharged early after surgery and the usability of continuous oxygen saturation devices for home telemonitoring. Additionally, this category encompasses specific safety programs, such as real-time remote patient monitoring for COVID-19 patients to manage isolation and detect deterioration.

The Prevention category aims to reduce disease risk through early screening and lifestyle interventions. This category is characterized by interventions such as mobile-assisted telehealth regimens to increase exercise in transplant candidates and technology-based weight management interventions for older adults. These studies align with the goals of preventative medicine by utilizing technology to support health behaviors and prevent adverse outcomes in high-risk populations.

The category of Rehabilitation centers on recovery and functional improvement after illness or injury. Research in this area explores systems such as portable remote rehabilitation following total knee replacement and web-based upper limb home rehabilitation systems using smartwatches for chronic stroke survivors. These interventions often employ wearable motion sensors or mobile applications to facilitate physical therapy and assess feasibility in a home setting.

Finally, Transitional Care ensures continuity and coordination of healthcare between different settings, particularly during the shift from hospital to home. Research in this group utilizes remote monitoring technologies to track health status upon discharge. This category encompasses programs conceptualized for patients with complex medical illness on hospital dismissal and research evaluating the effect of remote monitoring on discharge to home and rehospitalization rates. These programs aim to bridge the gap in care, ensuring patient safety and support during critical transition periods.

### Type of wearables

Regarding the types of wearables used, as evidenced by Table 13, 34 of the 55 studies reported using wristbands or smartwatches. Adhesives or patch sensors were used in seven studies, lower-limb sensors were used in four studies, namely two leg band sensors, two ankle-worn sensors, and two shoe-attached sensors, respectively, and rings were used in two studies. Additionally, some studies utilized more distinct sensor types: van den Bergh et al.^47^ utilized a necklace sensor, Wurzer et al.^15^ utilized an in-ear photoplethysmography (PPG) sensor, Chen et al.^56^ used a three-band sensor kit (wrist, arm and sternum worn), Wong et al.^40^ opted for an arm-band sensor, Sze et al.^44^ allowed participants to choose between a pocket-worn or a wrist band pedometer, Motolese et al.^42^ utilized an EEG head band, and Kikuchi et al.^45^ used a smart fabric shirt. The study by Charkviani et al.^58^ reported using an oximeter wearable but did not specify its design type.

**Table 13.** Types of wearables.

Type of wearable	References
Wristbands or smartwatches	Adams et al., (2022)^16^ Althobiani et al., (2023)^57^ Anand et al., (2021)^59^ Batsis et al., (2021)^17^ Blair et al., (2021)^73^ Chae et al., (2020)^51^ Davies et al., (2021)^50^ Ding et al., (2021)^70^ Downing et al., (2023)^71^ Duarte-Rojo et al., (2023)^75^ Fanning et al., (2020)^67^ Ha et al., (2022)^29^ Harzand et al., (2023)^72^ Hesketh et al., (2025)^32^ Indraratna et al., (2021)^24^ Khusial et al., (2020)^48^ LeBaron et al., (2023)^65^ Leitner et al., (2022)^12^ Leitner et al., (2024)^74^ Li et al., (2024)^55^ Liu et al., (2022)^33^ Loh et al., (2022)^60^ Mehta et al., (2020)^64^ O’Connor et al., (2025)^61^ Oftring et al., (2025)^36^ Patel et al., (2022)^13^ Sañudo et al., (2024)^52^ Stubberud et al., (2020)^49^ Sze et al., (2023)^44^ Toh et al., (2023)^41^ Toh et al., (2024)^39^ Tzeng et al., (2025)^53^ Wilmink et al., (2020)^62^ Wu et al., (2024)^31^
Adhesives or patch sensors	Blair et al., (2021)^73^ Breteler et al., (2020)^11^ Leenen et al., (2023)^30^ Matthews et al., (2024)^69^ Moore et al., (2025)^68^ Stubberud et al., (2020)^49^ van Ede et al., (2022)^46^
Lower-limb sensors	Bell et al., (2020)^63^ Cochen De Cock et al., (2021)^35^ Fanning et al., (2020)^67^ Gaßner et al., (2022)^38^
Rings	Bonometti et al., (2023)^43^ Purnell et al., (2023)^14^
Other sensors	Charkviani et al., (2023)^58^ Chen et al., (2020)^56^ Cochen De Cock et al. (2021)^35^ Fanning et al. (2020)^67^ Gaßner et al. (2022)^38^ Kikuchi et al., (2021)^45^ Klein et al. (2024)^37^ Li et al. (2025)^34^ Lin et al. (2024)^54^ Motolese et al., (2023)^42^ Sze et al., (2023)^44^ van den Bergh et al., (2023)^47^ Wong et al., (2022)^40^ Wurzer et al., (2021)^15^

The majority of these studies (n = 53) are employed commercially available wearable devices. Specifically, Fitbit was used in seven studies,^17,48,61,67,72,74,75^ Garmin in four,^53,57,59,60^ Apple Watch in two,^71,74^ Phillips in two,^40,62^ Xiaomi in two,^44,45^ and Samsung in one.^12^ The remaining studies (n = 34) used other brands or devices not clearly linked to major commercial manufacturers. Notably, 4 out of the 55 studies^37,39,62,69^ analyzed clearly reported using dedicated, self-developed wearables in their studies, while the study by Toh et al.^41^ did not specify the origin of the wearable sensor used.

The heavy focus on wristbands and smartwatches across studies suggests a solid, effective approach to wearable monitoring, establishing their reliability, and ease of use. Other sensor types, such as adhesive-based, rings or smart fabrics are utilized, but there remains an opportunity to more deeply explore these other wearable designs, which may offer advantages in specific scenarios or populations. Regarding device origin, the reliance on previously available devices like Fitbit and Garmin appears sufficient for most research needs, highlighting the practicality of leveraging existing technology, benefiting studies by reducing development time and costs, while still providing reliable data.

### Data collection

When analyzing the types of data collected as reported by the review studies, a clear distinction emerged between data directly obtained from wearable sensors and data derived from it, leading to their separate categorization in this section.

Among the 55 studies, various types of data directly collected from wearable sensors were reported. The most common type, as clearly shown in Table 14, was Inertial measurement unit data, namely limb or body acceleration, orientation, and angular velocity (n = 17). Other types of direct data reported included body temperature (n = 8), PPG data (n = 4), and electrocardiography (ECG) data (n = 5). In 17 studies, the type of sensor data collected was not specified.

**Table 14.** Most common type of data collected reported.

Most common type of data collected	References
IMU	Adams et al., (2022)^16^ Bell et al., (2020)^63^ Blair et al., (2021)^73^ Chae et al., (2020)^51^ Chen et al., (2020)^56^ Cochen De Cock et al., (2021)^35^ Gaßner et al., (2022)^38^ Ha et al., (2022)^29^ Leitner et al., (2022)^12^ Motolese et al., (2023)^42^ Purnell et al., (2023)^14^ Sañudo et al., (2024)^52^ Toh et al., (2023)^41^ Toh et al., (2025)^39^ van den Bergh et al., (2023)^47^ van Ede et al., (2022)^46^ Wilmink et al., (2020)^62^
Body temperature	Breteler et al., (2020)^11^ Lin et al., (2024)^54^ Matthews et al., (2024)^69^ Moore et al., (2025)^68^ Oftring et al., (2025)^36^ Walter et al., (2023)^66^ Wong et al., (2022)^40^ Wurzer et al., (2021)^15^
PPG	Matthews et al., (2024)^69^ Motolese et al., (2023)^42^ Patel et al., (2022)^13^ Wurzer et al., (2021)^15^
ECG	Matthews et al., (2024)^69^ Stubberud et al., (2020)^49^ Kikuchi et al., (2021)^45^ Klein et al., (2024)^37^ Liu et al., (2022)^33^

PPG: photoplethysmography; IMU: Inertial measurement unit; ECG: electrocardiography.

Regarding data derived from wearables, all studies (n = 55) reported processed outputs, including physiological signals, vital signs, or ML-derived metrics. The most common, present in Table 15, were heart rate (n = 28), step count (n = 20), oxygen saturation (SpO2) (n = 13), respiratory rate (n = 12), and physical activity levels (n = 13).

**Table 15.** Most common data derived reported.

Most common data derived	References
Heart rate	Althobiani et al., (2023)^57^ Bonometti et al., (2023)^43^ Breteler et al., (2020)^11^ Charkviani et al., (2023)^58^ Davies et al., (2021)^50^ Downing et al., (2023)^71^ Harzand et al., (2023)^72^ Indraratna et al., (2021)^24^ Khusial et al., (2020)^48^ Kikuchi et al., (2021)^45^ Klein et al., (2024)^37^ Leenen et al., (2023)^30^ Leitner et al., (2022)^12^ Leitner et al., (2024)^74^ Li et al., (2024)^55^ Matthews et al., (2024)^69^ Moore et al., (2025)^68^ Motolese et al., (2023)^42^ O'Connor et al., (2025)^61^ Oftring et al., (2025)^36^ Patel et al., (2022)^13^ Purnell et al., (2023)^14^ Stubberud et al., (2020)^49^ van Ede et al., (2022)^46^ Walter et al., (2023)^66^ Wong et al., (2022)^40^ Wu et al., (2024)^31^ Wurzer et al., (2021)^15^
Step count	Althobiani et al., (2023)^57^ Anand et al., (2021)^59^ Batsis et al., (2021)^17^ Blair et al., (2021)^73^ Breteler et al., (2020)^11^ Cochen De Cock et al., (2021)^35^ Davies et al., (2021)^50^ Ding et al., (2021)^70^ Duarte-Rojo et al., (2023)^75^ Fanning et al., (2020)^67^ Harzand et al., (2023)^72^ Khusial et al., (2020)^48^ Leitner et al., (2022)^12^ Leitner et al., (2024)^74^ Li et al., (2024)^55^ Loh et al., (2022)^60^ O'Connor et al., (2025)^61^ Sañudo et al., (2024)^52^ Sze et al., (2023)^44^ Wong et al., (2022)^40^
Oxygen saturation	Althobiani et al., (2023)^57^ Bonometti et al., (2023)^43^ Charkviani et al., (2023)^58^ Indraratna et al., (2021)^24^ Klein et al., (2024)^37^ Matthews et al., (2024)^69^ Motolese et al., (2023)^42^ O'Connor et al., (2025)^61^ Patel et al., (2022)^13^ Walter et al., (2023)^66^ Wong et al., (2022)^40^ Wu et al., (2024)^31^ Wurzer et al., (2021)^15^
Respiratory rate	Althobiani et al., (2023)^57^ Breteler et al., (2020)^11^ Leenen et al., (2023)^30^ Matthews et al., (2024)^69^ Moore et al., (2025)^68^ Motolese et al., (2023)^42^ Oftring et al., (2025)^36^ Patel et al., (2022)^13^ van Ede et al., (2022)^46^ Walter et al., (2023)^66^ Wong et al., (2022)^40^ Wurzer et al., (2021)^15^
Physical activity levels	Anand et al., (2021)^59^ Ha et al., (2022)^29^ Indraratna et al., (2021)^24^ LeBaron et al., (2023)^65^ Leenen et al., (2023)^30^ Leitner et al., (2022)^12^ Liu et al., (2022)^33^ Oftring et al., (2025)^36^ Sañudo et al., (2024)^52^ van den Bergh et al., (2023)^47^ Walter et al., (2023)^66^ Wilmink et al., (2020)^62^ Wu et al., (2024)^31^

The diversity of data types observed, both collected and derived, shows the broad applicability of wearable technology and RHM across various fields of research and clinical practice. The emphasis on physiological and activity-related metrics suggests a strong focus on cardiovascular and physical health monitoring as the most sought-after fields.

Meanwhile, a predominance of reported derived data over raw sensor data could be observed in the reviewed studies. While direct access to raw data could provide additional analytical opportunities, its omission in nearly half the studies may reflect the fact that processed outputs are often sufficient for both healthcare professionals and researchers conducting remote monitoring. This reliance on derived metrics may indicate that wearable devices are successfully designed to minimize complexity for end users while still offering valuable health insights.

### Data visualization and user interfaces

Visualization solutions to present information to patients and healthcare workers varied widely across the selected studies, which considered a combination of mobile applications, web dashboards, and specialized platforms. Overall, these solutions aimed to enhance patient engagement with treatment and physical activity plans, enabling real-time monitoring and facilitating communication between patients and healthcare teams.

In 30 studies,^14,24,29,30,32–34^^,36,39,41,43–52,55–57,59,61–63,69–71^ participants were able to access their health data through mobile applications installed in smartphones or tablets. These apps allowed the participants to monitor their health data such as heart rate, blood oxygen levels, temperature, and physical activity. In some cases, apps provide real-time feedback, including visualizations of daily progress, gait performance, and session statistics, focusing on easy-to-understand visual aids, such as progress bars, thresholds, and real-time status updates. For chronic conditions or rehabilitation, some applications included educational videos, exercise reminders and daily progress tracking, encouraging participants to engage with their treatment plans. In some cases, participants only required a phone to visualize their performance through text messages.^16^ Unlike other cases, the study of Blair et al.,^73^ participants had access to their data, but it was processed by researchers who generated summary graphs of their most and least active days and later shared them via email for review with their coach.

Healthcare professionals often had access to dedicated web-based platforms or application dashboards, with 32 studies,^11,13,15,17,29–31^^,34,37,40,42,43,45,46,48,51,54,56–63,65,66,68–70,73,75^ in this review reporting their use. In fact, 20 out of 32 studies adopted such web applications for data visualization for healthcare providers, rather than mobile applications.^15,17,29–31^^,34,37,40,42,43,45,46,48,51,54,57,63,66,69,70^ These dashboards provided comprehensive overviews of patient data, including alerts for abnormal readings and detailed charts of physiological parameters. Data visualization platforms were used to analyze patterns in physical activity, staff efficiency, and resident care metrics, enhancing overall health management.^31^ Other studies, distinct from those previously mentioned, do not utilize web-based dashboards, platforms, or apps. Instead, healthcare professionals access users’ data through screenshots of reports sent by each user via email or text message^14^ or retrieve stored and processed data from cloud-based systems.^52^ In this review, eight studies^12,16,35,38,53,64,72,74^ do not provide data visualization for either patients or healthcare professionals.

The wide range of data visualization tools within the systems suggests the importance of accessible and user-friendly interfaces for both patients and healthcare professionals. Mobile applications providing real-time feedback and educational content have shown efficacy in promoting behavior change. For healthcare professionals, dashboards are indispensable for summarizing and interpreting patient data. Most of the studies adopted such web applications for data visualization for healthcare providers, rather than mobile applications. The preference for web-based dashboards or platforms among healthcare professionals likely may reflect their practicality in most clinical scenarios and workflows. These solutions may offer advantages in security, scalability, integration with EHR systems and administrative tools, and feasibility for handling intensive data processing, such as large datasets and complex visualizations. For patients, mobile applications were a commonly used data visualization resource among the reviewed studies, not only due to widespread use of smartphones globally and their capacity to integrate with wearable devices via Bluetooth, but also due to convenience in involving patients in the treatment (e.g. portability, interactivity), presenting patient information in real time and enabling the usage of alerts and recommendations.

### Feedback system

Out of the 55 studies reviewed, 49 have some type of feedback system (to patient or healthcare professional, or both) that varied significantly. Table 16 presents the feedback system orientation, and the type of feedback used in the 49 studies.

**Table 16.** Feedback system orientation and feedback type.

Feedback orientation	Feedback type	References
Patients	Automatic	Adams et al., (2022)^16^ Althobiani et al., (2023)^57^ Bell et al., (2020)^63^ Blair et al., (2021)^73^ Cochen De Cock et al., (2021)^35^ Davies et al., (2021)^50^ Ding et al., (2021)^70^ Leitner et al., (2022)^12^ Motolese et al., (2023)^42^ Purnell et al., (2023)^14^ Sañudo et al., (2024)^52^ Stubberud et al., (2020)^49^ Sze et al., (2023)^44^ Toh et al., (2023)^41^ van den Bergh et al., (2023)^47^ Wong et al., (2022)^40^ Wu et al., (2024)^31^ Fanning et al., (2020)^67^ Hesketh et al., (2025)^32^ Leenen et al., (2024)^30^ Leitner et al., (2024)^74^ Li et al., (2025)^34^ Liu et al., (2022)^33^ Mehta et al., (2020)^64^ Oftring et al., (2025)^36^ Toh et al., (2025)^39^ Tzeng et al., (2025)^53^
	Non-automatic	Anand et al., (2021)^59^ Batsis et al., (2021)^17^ Chae et al., (2020)^51^ Charkviani et al., (2023)^58^ Chen et al., (2020)^56^ Duarte-Rojo et al., (2023)^75^ Gaßner et al., (2022)^38^ Harzand et al., (2023)^72^ Kikuchi et al., (2021)^45^ Li et al., (2024)^55^ Loh et al., (2022)^60^ Wilmink et al., (2020)^62^ Wurzer et al., (2021)^15^ Moore et al., (2025)^68^
Healthcare professionals	Automatic	Althobiani et al., (2023)^57^ Bonometti et al., (2023)^43^ Davies et al., (2021)^50^ Downing et al., (2023)^71^ Indraratna et al., (2021)^24^ Matthews et al., (2024)^69^ van Ede et al., (2022)^46^ Wilmink et al., (2020)^62^ Leenen et al., (2024)^30^ Li et al., (2025)^34^ Lin et al., (2024)^54^ Moore et al., (2025)^68^ O’Connor et al., (2025)^61^ Walter et al., (2023)^66^
	Non-automatic	-

Among these studies, 41 provided feedback to the patient with 27 providing automatic feedback, including data summaries,^14,73^ alerts for deviations from the protocol of the study,^57^ reminders to encourage adherence to medication,^70^ or physical activity adjustments,^48,56^ while the remaining studies (n = 14) delivered feedback through methods such as calls and reports.

Only 14 studies provided automatic feedback to healthcare professionals in case of participant deterioration or abnormal health signs, per example, so that they can adjust treatment plans and provide individualized recommendations based on participant real-time data.

Overall, the diversity in feedback mechanisms illustrates the potential of remote monitoring systems to enhance and personalize patient care, showing how tailored feedback can improve patient engagement and adherence. However, many studies lack transparency about the behavioral or motivational strategies that drive these feedback systems. This limited information restricts the replication of effective strategies in other research, potentially slowing progress in this field. By more thoroughly documenting and sharing the design and theoretical basis of feedback mechanisms, future studies could foster more consistent improvements in patient engagement and health outcomes across different applications.

### Machine learning

Most studies (n = 47) did not employ or explicitly mention ML, focusing instead on traditional statistical methods or observational data analysis. In some cases, ML models were neither applicable nor relevant to the study's design, particularly in early development or proof-of-concept stages.

A few studies (n = 8) employed specific ML methods for data analysis, prediction, and personalized recommendations, focusing mostly on sensor analytics, whereas Moore et al.^68^ investigated the use of ML to improve communication and triage in a remote monitoring system. In this research, an AI-enabled chatbot was implemented to respond to patient-reported symptoms, as well as to adapt messages sent by patients in order to provide review-of-systems queries representing standard medical assessments.

Convolutional neural networks (CNN) were used in some studies for complex pattern recognition tasks. Chae et al.^51^ trained a CNN model to detect and classify home exercises performed by participants, requiring the creation of a custom dataset where participants repeated exercises multiple times while wearing a smartwatch. The dataset was used to train both individual models (specific to each participant) and a generalized model (using data from all participants). Matthews et al.^69^ employed a Residual CNN model to predict systolic and diastolic blood pressure, demonstrating the capability of CNNs in handling physiological data beyond image processing tasks.

ML has also been applied to the analysis of specific physical parameters,^38^ particularly in the estimation of spatiotemporal gait parameters such as stride length and gait velocity. Although the specific details of the models were not disclosed, this approach emphasizes the potential of ML in movement pattern analysis for clinical assessments and rehabilitation. Additionally, Leitner et al.^12^ used ML to generate personalized lifestyle recommendations based on a 30-day rolling window of participant data. Similarly, Li et al.^34^ employed AI-driven algorithms to tailor exercise prescriptions and adjust training intensity based on ongoing patient data. Moreover, Motolese et al.^42^ utilized signal processing and ML techniques to analyze brain signal components, enabling real-time control of auditory experiences.

The limited use of ML algorithms likely reflects the exploratory nature of many studies, where the focus was on feasibility or observational insights rather than predictive modeling. However, studies that applied deep learning methods, namely CNNs, have demonstrated significant potential in health monitoring and prediction. Chae et al.’s^51^ use of CNNs for exercise classification aligns with broader applications of such algorithms in personalized fitness recommendations and physical therapy. Additionally, the use of AI for producing personalized interventions, as explored by Leitner et al.,^74^ reflects broader trends in personalized medicine and is expected to reshape healthcare by tailoring treatments to individual needs, improving outcomes, and contributing to patient-centered care.

It is important to note that detailed technical specifications, such as validation schemes (e.g. k-fold cross-validation) and performance metrics (e.g. accuracy, sensitivity), were frequently underreported or omitted in these clinically oriented studies, limiting the ability to conduct a deeper technical comparison.

### Main findings

The feasibility and acceptance of remote monitoring and telerehabilitation are widely supported across the literature, though implementation nuances significantly impact engagement. Althobiani et al.^57^ and Oftring et al.^36^ established that remote monitoring is feasible and well-accepted, particularly when utilizing passive data collection, although technical connectivity issues remain a challenge. Leenen et al.^30^ reinforced this, reporting sufficient usability ratings for platform-based monitoring, while Moore et al.^68^ found that patients and caregivers perceived these systems as making them feel “more cared for,” despite a desire for better alerting processes. This sense of security was echoed by Wurzer et al.,^15^ whose participants felt safer at home with wireless patch sensors, enabling earlier discharge. User satisfaction often translated into clinical utility; Lin et al.^54^ noted that abnormal readings successfully triggered interventions without adverse events, and Ding et al.^70^ highlighted high usability for blood pressure apps, though patients were hesitant to share data.

These patient-centric benefits scale up to significant system-level efficiencies. Charkviani et al.^58^ and Downing et al.^71^ concluded that RPM facilitates the transition to home and effectively reduces readmission rates. Indraratna et al.^24^ supported this, noting that 97% of alerts were managed in outpatient settings, preventing hospitalization. Similarly, Mehta et al.^64^ observed reduced rehospitalization rates in intervention groups, while Walter et al.^66^ quantified the economic impact, identifying a 12% reduction in length of stay and substantial cost savings per patient. Even when costs remained neutral, as Bell et al.^63^ found, the reduction in physical therapy visits and high willingness to reuse the system demonstrated operational value.

In the management of cardiovascular health, remote interventions have driven measurable clinical improvements. Leitner et al.^12^ and Leitner et al.^74^ demonstrated that remote programs significantly improved blood pressure regulation with minimal clinician intervention. Beyond hypertension, Wong et al.^40^ showed that monitoring facilitated the optimization of heart failure medications, specifically increasing the dosages of ARNI and MRA therapies. Liu et al.^33^ found that smartwatches could detect 76% of cardiac events to support decision-making, while Matthews et al.^69^ validated similar systems for high-risk postpartum women. In the context of cardiac rehabilitation, Kikuchi et al.,^45^ Harzand et al.,^72^ and Tzeng et al.^53^ all reported significant increases in 6-minute walk test distances and reductions in risk factors like smoking and cholesterol, proving telerehabilitation to be a viable alternative to center-based care.

Similar success has been observed in respiratory and post-surgical monitoring, where Bonometti et al.^43^ found that tracking oxygen saturation reduced hospital visits. Khusial et al.^48^ reported that the myAirCoach system reduced severe asthma exacerbations, and Patel et al.^13^ confirmed the utility of RPM for managing oxygen therapy in post-discharge COVID-19 patients. While O'Connor et al.^61^ noted improvements in assessment scores for COPD, Wu et al.^31^ highlighted a limitation, finding that smartwatch apps alone without a comprehensive care program did not improve self-management. In surgical contexts, Breteler et al.^11^ found remote monitoring after esophagectomy supported clinical judgment, and van Ede et al.^46^ demonstrated that wearables could detect complications after bariatric surgery with 75% sensitivity.

Technological interventions have also shown efficacy in neurological recovery. Chae et al.^51^ and Chen et al.^56^ found that integrating sensors and ML into stroke rehabilitation improved range of motion and functional scores compared to standard home exercises. Toh et al.^41^ and Toh et al.^39^ further validated wearable-assisted telerehabilitation, noting high compliance and superior improvements in shoulder range of motion compared to conventional therapy. For Parkinson's disease, Cochen De Cock et al.^35^ and Gaßner et al.^38^ observed improvements in gait parameters and motor tasks of daily living, respectively, with van den Bergh et al.^47^ adding that personalization is key to acceptance among physiotherapists. Additionally, Davies et al.^50^ found mHealth feasible for monitoring pediatric epilepsy, capturing data more effectively than paper diaries despite socioeconomic barriers.

Broader lifestyle and metabolic interventions have yielded mixed but generally positive outcomes. Anand et al.^59^ and Batsis et al.^17^ found remote interventions effective for enhancing physical activity and weight loss across diverse populations. Fanning et al.^67^ reported meaningful improvements in physical function and pain through the MORPH intervention, while Sze et al.^44^ and Hesketh et al.^32^ noted increased steps and improved HbA1c in diabetes management. However, increasing activity does not always equate to reduced sedentary time, as noted by Blair et al.^73^ and Sañudo et al.,^52^ who found that while steps increased, sleep and sedentary habits remained largely unchanged. Addressing engagement issues, Adams et al.^16^ proved that adaptive goals with immediate reinforcement were significantly more effective than static goals.

Finally, the scope of remote monitoring extends effectively to specialized populations and mental health applications. Ha et al.^29^ and Li et al.^34^ found these interventions feasible for cancer survivors, resulting in reduced anxiety and fatigue, while Loh et al.^60^ and Li et al.^55^ established feasibility and physical benefits for older adults undergoing chemotherapy and those with kidney disease, respectively. For geriatric safety, Wilmink et al.^62^ showed that the CarePredict system reduced falls, and Duarte-Rojo et al.^75^ utilized wearables to improve frailty indices in liver patients. In the mental health domain, Klein et al.^37^ found wearables helpful in treating OCD, Motolese et al.^42^ showed that meditative exercises improved cognitive function, and Stubberud et al.^49^ validated biofeedback apps. While LeBaron et al.^65^ successfully used ecological momentary assessments to capture pain patterns, Purnell et al.^14^ noted that while devices like the SomnoRing are easy to use, users ultimately require more actionable insights from their data to drive behavioral change.

### Quality assessment

**Figure 7.**
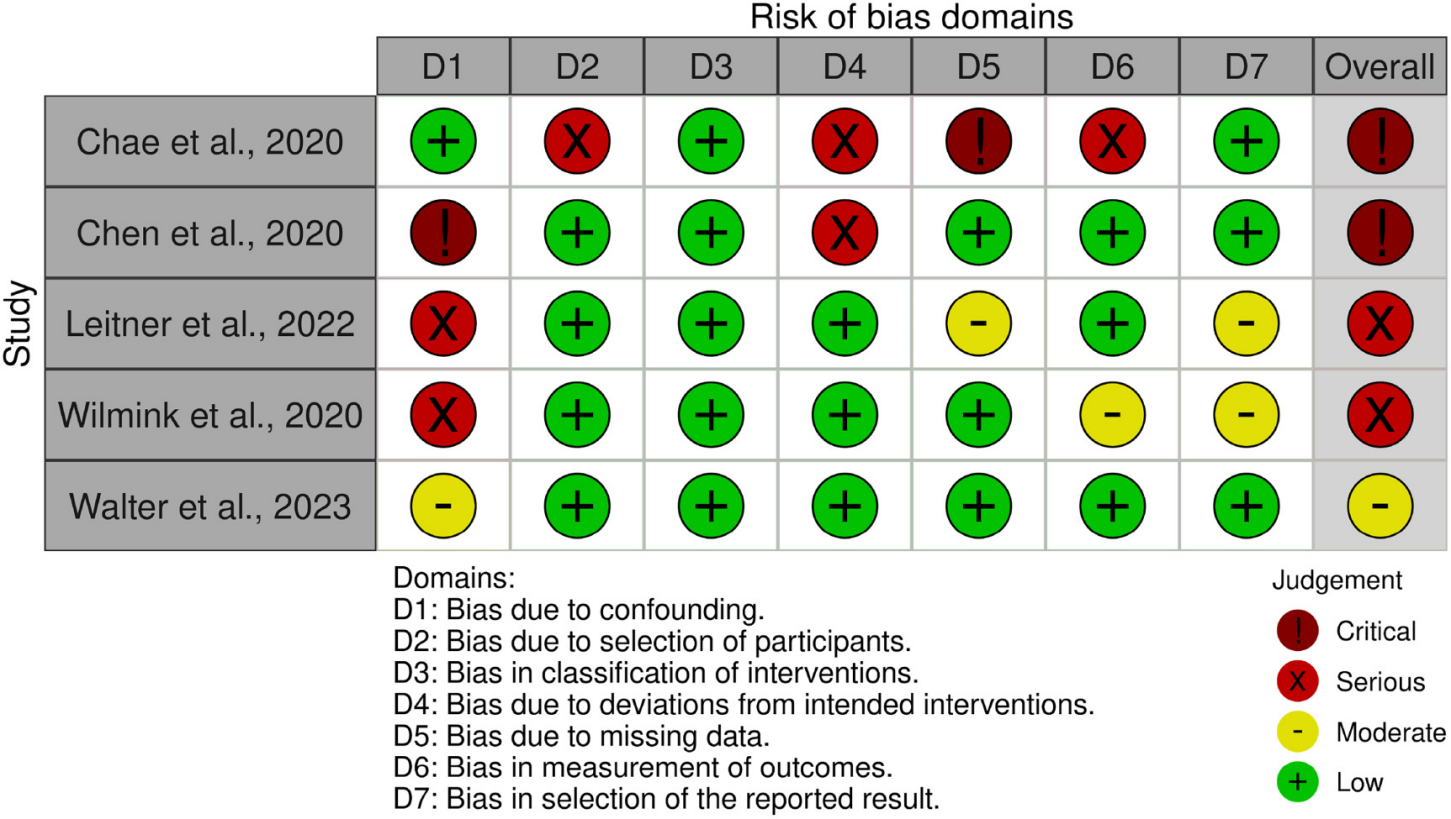
Risk of bias of non-randomized controlled trials according to ROBINS-I. ROBINS-I: Risk of Bias In Non-randomized Studies of Interventions

The risk of bias analysis revealed marked variability in methodological quality across the included studies, shown in Figure 7. Among the five non-randomized studies assessed with the ROBINS-I tool, four were judged as having an overall serious or critical risk of bias, mainly due to confounding, deviations from intended interventions, and missing data. Only one study reached a moderate overall rating, with low risk across most domains but some remaining limitations. Notably, none of the studies evaluated with ROBINS-I achieved a low risk of bias, possibly indicating substantial methodological challenges inherent to these observational or quasi-experimental designs.

**Figure 8.**
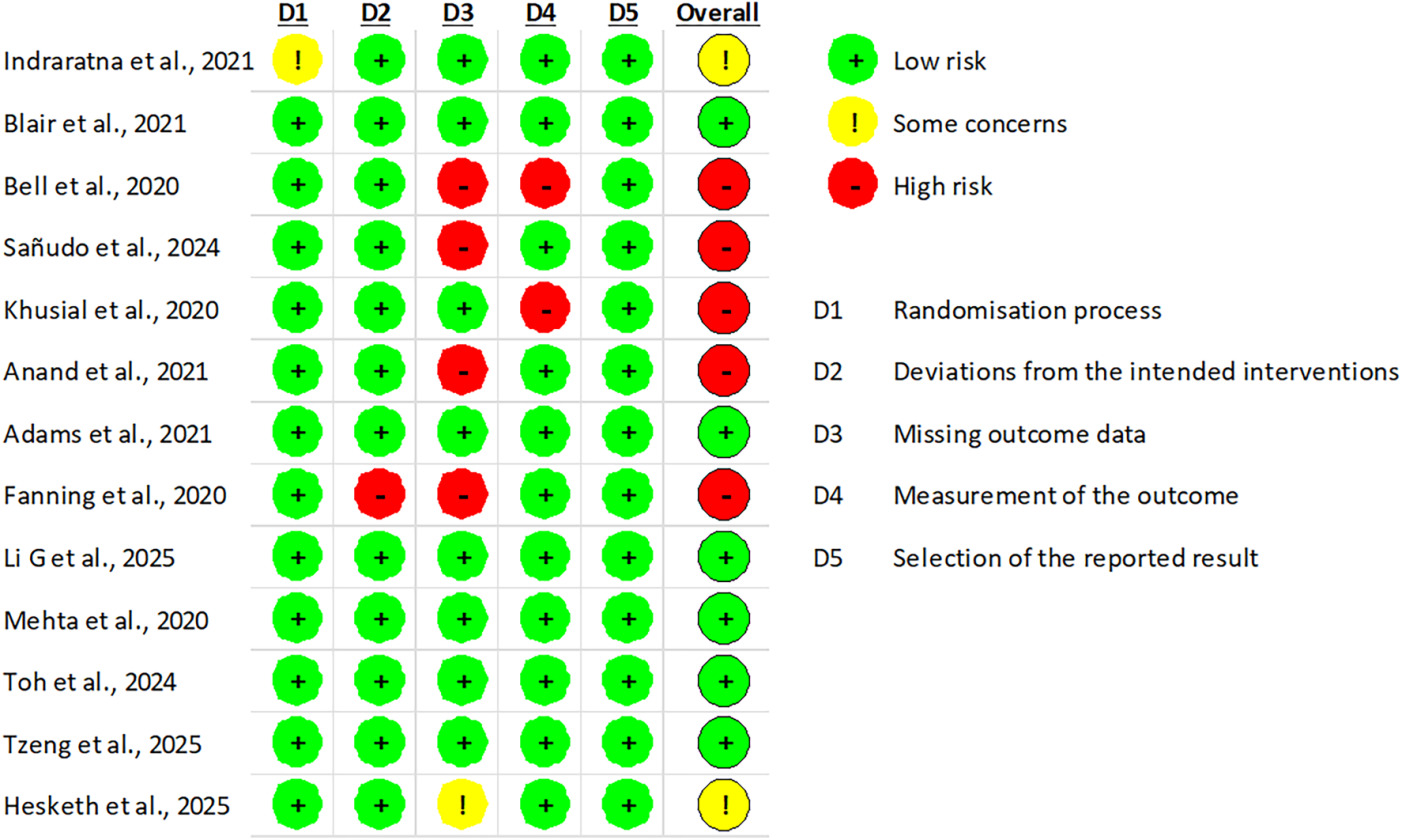
Risk of bias of randomized controlled trials according to RoB2.RoB2: Risk of Bias 2.

In contrast, the 13 RCTs evaluated using RoB 2 (Figure 8) demonstrated comparatively stronger methodological quality. Within this group, six trials were rated as having an overall low risk of bias, with consistently low judgments across domains such as randomization, deviations from intended interventions, and outcome measurement. However, two studies were classified as having some concerns, typically due to issues related to randomization process and missing outcome data. Additionally, five trials were judged to be at high risk of bias, mainly due to problems in missing outcome data and measurement of the outcome.

## Discussion

In this section, we critically examine the findings presented in the results chapter to interpret the current trajectory of wearable-based RHM. By analyzing the data synthesized from the 55 selected studies, we can identify prevailing trends and outline critical directions for future research. The substantial volume of literature published between 2020 and 2025 underscores a rapid expansion in this field, driven by technological advancements and the shifting needs of domiciliary care. While this growth indicates a rising interest in deploying these solutions, a closer look at the results reveals significant disparities in technological application and clinical focus that warrant further discussion.

RQ1 – What are the main wearable technologies employed in the field of remote monitoring?

The landscape of remote monitoring is currently dominated by wrist-worn devices, with 34 of the 55 reviewed studies utilizing smartwatches or fitness trackers. This prevalence highlights a heavy reliance on commercially available technology, such as Fitbit and Garmin devices, rather than custom-built hardware. This trend likely reflects a pragmatic choice by researchers to leverage the usability, battery life, and established data ecosystems of consumer-grade devices, which reduces development costs and deployment friction. However, this reliance creates a “black box” limitation regarding the depth of clinical data available. Nearly half of the studies relied on derived metrics, such as step counts (n = 20) or sleep scores, rather than processing raw sensor data like accelerometer or PPG signals. While utilizing pre-processed metrics simplifies the analytical workflow, it significantly restricts the ability to develop novel biomarkers or conduct deep signal analysis that raw data would permit. Furthermore, alternative form factors remain underexplored; adhesive patches and smart textiles were employed in only a handful of studies (n = 7 and n = 1, respectively). This is a critical gap, as wrist-worn devices may not be suitable for all populations (e.g. those with tremors or skin integrity issues) or for capturing specific metrics like core body temperature or high-fidelity ECG, where patches often offer superior accuracy and continuous contact.

RQ2 – Which categories dominate the use of wearable technologies for remote patient monitoring?

The application of wearable technologies is heavily skewed towards the management of chronic conditions and the care of older adults. Our categorization shows that “Monitoring” (n = 24) and “Rehabilitation” (n = 14) are the primary focus areas. Clinically, this translates to a strong emphasis on cardiovascular and respiratory diseases, likely because these conditions align well with the standard metrics captured by current wearables, such as heart rate and oxygen saturation. A critical observation regarding participant demographics is the distinct age gap in the literature: the vast majority of research targets individuals aged 50 to 75, with a complete absence of studies focusing on adults in the 30–40 age range and only one in the 20–30 range. This suggests that remote monitoring is currently conceptualized primarily as a reactive tool for aging populations or established disease management, rather than a preventative strategy for the broader working-age population. Furthermore, while physical health domains are well-represented, there is a notable scarcity of research addressing cognitive and psychological well-being. Only a few studies, such as those by Klein et al.^37^ on OCD and Motolese et al.^42^ on meditative exercises, ventured into mental health, indicating a significant missed opportunity to utilize wearables for detecting physiological correlates of stress, anxiety, or cognitive decline.

RQ3 – What are the most frequent feedback strategies/mechanisms considered in the context of remote health monitoring?

Feedback systems are integral to these interventions, present in 49 studies, yet they reveal a significant imbalance in orientation. The strategies are overwhelmingly patient-centric, with 41 studies providing feedback directly to the user. This feedback is predominantly operational (consisting of automatic alerts, medication reminders, and data summaries) designed to enhance adherence and engagement. While mobile applications serve as an effective interface for this visualization, a major limitation is the lack of transparency regarding the theoretical basis of these feedback loops. Few studies explicitly link their mechanisms to behavioral change theories, resulting in feedback that is often generic rather than personalized or psychologically grounded. This may lead to “notification fatigue,” where users become desensitized to constant, non-adaptive alerts. Conversely, automatic feedback for healthcare professionals is rare, reported in only 14 studies. Most systems provide data to clinicians via passive web dashboards, which relies on the clinician to actively log in and check data. This “pull” model misses the opportunity to implement automated “push” systems (triage or early-warning algorithms) that could support decision-making without contributing to administrative burden or requiring constant surveillance by the medical team.

RQ4 – Which artificial intelligence methods are most used in the context of remote health monitoring?

Despite the theoretical promise of AI in transforming healthcare data into actionable insights, its practical application in remote monitoring remains limited and largely exploratory. The vast majority of studies (47 out of 55) relied on traditional statistical methods or observational analysis, with only eight studies integrating ML techniques. Where AI was employed, it showed significant potential to move beyond simple monitoring. For instance, CNN were successfully used by Chae et al.^51^ to classify specific home rehabilitation exercises, and by Matthews et al.^69^ to predict blood pressure from cuffless sensors. These examples demonstrate AI's capacity to act as a “virtual coach” or a diagnostic tool, rather than just a data logger. However, the scarcity of these advanced methods suggests that the field is still in a data collection phase rather than an analytical one. The lack of large, labeled datasets and the complexity of validating “black box” algorithms in clinical settings likely hinder broader adoption. To transition from passive monitoring to precision medicine, future research must prioritize the collection of high-quality, raw sensor data to train predictive models that can anticipate adverse events before they manifest clinically.

Beyond the specific research questions, the risk of bias assessment revealed marked variability in the methodological quality of the evidence base. Among the non-randomized studies evaluated with the ROBINS-I tool, the majority were judged to have a serious or critical risk of bias, primarily due to confounding factors, deviations from intended interventions, and missing data. No observational study achieved a low risk of bias, reflecting the inherent difficulties in controlling variables within real-world home monitoring contexts. In contrast, RCTs demonstrated comparatively stronger quality; six trials were rated as having a low overall risk of bias using the RoB 2 tool, although issues regarding missing outcome data persisted in some studies. This heterogeneity in evidence quality necessitates caution when interpreting the effectiveness of these interventions and underscores the urgent need for more rigorous, standardized research designs in future work.

Furthermore, the large-scale implementation of these technologies faces significant regulatory and operational hurdles. A critical barrier is the distinction between consumer-grade wearables and medical-grade devices, which complicates regulatory approval and reimbursement processes. While some studies demonstrated that remote monitoring could reduce hospital length of stay and costs, universal reimbursement models are not yet established, limiting economic sustainability. Data privacy also emerges as a paramount concern, with patients frequently expressing hesitancy to share sensitive health data, highlighting the need for robust encryption and strict compliance with data protection frameworks. Finally, successful implementation relies on digital equity and provider acceptance. Systems must integrate seamlessly into electronic health records to provide actionable insights without causing “alert fatigue” among clinicians, while simultaneously ensuring that socioeconomic barriers do not exclude vulnerable populations from accessing these digital health innovations.

## Limitations

Several limitations should be considered when interpreting the findings of this review. First, although the screening and data extraction processes involved more than one reviewer to reduce subjectivity, human error cannot be fully excluded. In later stages of the review, when fewer reviewers were involved, the potential for inaccuracies in data extraction and classification may have increased.

Second, the review includes studies published up to October 2025. Given the rapid evolution of remote monitoring technologies and the continuous release of new sensor-based solutions, relevant studies published after this date may not have been captured. Additionally, some recently completed studies may not yet have been indexed in major databases at the time of the search.

Third, the included studies themselves present several methodological limitations. Many had small sample sizes, short intervention durations, or heterogeneous populations, which limited the robustness and comparability of outcomes. Selection bias was also common, as most interventions required participants to have access to digital technologies and a certain level of technological literacy, restricting generalizability to the broader population. In several studies, the absence of a comparator group, the inability to blind participants, and the presence of uncontrolled confounding variables may have influenced the reported effects. These issues were reflected in the risk-of-bias assessment, where most non-randomized studies demonstrated serious to critical risks of bias.

Fourth, although data extraction was standardized, the considerable heterogeneity in intervention types, monitoring contexts, sensor modalities, and outcome measures posed challenges in achieving complete uniformity. This diversity rendered a quantitative meta-analysis methodologically inappropriate, as pooling data across such disparate studies would yield invalid conclusions. Similarly, due to the high heterogeneity of clinical domains and the limited number of studies within specific sub-categories, it was not feasible to perform a robust stratified analysis of evidence strength across disease types or device forms. Consequently, a narrative synthesis was adopted to best capture the breadth of the current state-of-the-art. Finally, despite efforts to synthesize the evidence as objectively as possible, some degree of subjectivity is inherent to narrative synthesis. The overall conclusions of this review depend on the quality, completeness, and reporting standards of the primary studies, which varied considerably across the included literature. Nonetheless, the review highlights important patterns and recurring features of remote monitoring interventions, offering valuable insights for future research and development.

## Conclusions and future directions

Remote patient monitoring has become an increasingly relevant approach for supporting continuous and non-invasive health assessment, largely driven by the widespread adoption of wearable sensors. This review synthesized evidence from studies published between 2020 and October 2025, identifying 55 eligible articles and examining them through four research questions focused on the technologies employed, the predominant application domains, the feedback strategies implemented, and the use of ML methods.

The findings indicate that wrist-worn devices, particularly smartwatches and fitness trackers, dominate current remote monitoring interventions, while adhesive-based sensors and alternative form factors remain underexplored despite their potential advantages for specific clinical or demographic groups. The predominance of commercially available devices, such as Fitbit and Garmin systems, highlights both the maturity and accessibility of these technologies, allowing researchers to leverage validated hardware without the cost and complexity of developing custom sensors.

The review further revealed that feedback strategies are predominantly patient-oriented, with automatic alerts, reminders, and data summaries widely adopted to promote engagement and adherence. However, clinician-directed feedback was comparatively rare, underscoring an opportunity to strengthen the integration of healthcare professionals into remote monitoring pathways.

Despite the increasing interest in intelligent systems, most studies relied on conventional statistical methods rather than ML techniques. Only a limited number incorporated algorithms such as CNNs, yet these instances demonstrated promising potential for predictive monitoring and early detection of adverse events. This suggests a meaningful opportunity to expand ML integration in future remote monitoring systems, particularly as larger datasets and more sophisticated sensing modalities become available.

Overall, the findings suggest that wearable-enabled remote monitoring shows promise in small-scale or early-stage trials. However, the evidence remains heterogeneous, and the overall quality is limited, highlighting several gaps that merit attention. The variability in study designs, short intervention durations, and limited adoption of advanced analytical methods illustrate the need for more rigorous, long-term, and methodologically robust research.

To advance the field beyond feasibility pilots, future research should adopt hybrid implementation-effectiveness designs that simultaneously evaluate clinical outcomes and real-world adoption hurdles. Researchers must also prioritize rigorous standardized reporting guidelines to ensure that methodological protocols, device specifications, and technical parameters are transparent and reproducible regardless of the technology used. Furthermore, feedback mechanisms should be explicitly grounded in proven behavioral change theories rather than generic alerts, and study durations must be extended (e.g. >6 months) to accurately assess the long-term sustainability of patient engagement and health benefits.

## Supplemental Material

sj-docx-1-dhj-10.1177_20552076261428387 - Supplemental material for A systematic review on wearable-enabled remote health monitoring Supplemental material, sj-docx-1-dhj-10.1177_20552076261428387 for A systematic review on wearable-enabled remote health monitoring by Rita Ribeiro, Rafael Martins, Hugo Pereira, Vítor Crista, Júlio Souza, Rute Almeida, Diogo Martinho, Luís Conceição, Alberto Freitas and Goreti Marreiros in DIGITAL HEALTH

sj-xlsx-2-dhj-10.1177_20552076261428387 - Supplemental material for A systematic review on wearable-enabled remote health monitoring Supplemental material, sj-xlsx-2-dhj-10.1177_20552076261428387 for A systematic review on wearable-enabled remote health monitoring by Rita Ribeiro, Rafael Martins, Hugo Pereira, Vítor Crista, Júlio Souza, Rute Almeida, Diogo Martinho, Luís Conceição, Alberto Freitas and Goreti Marreiros in DIGITAL HEALTH

sj-docx-3-dhj-10.1177_20552076261428387 - Supplemental material for A systematic review on wearable-enabled remote health monitoring Supplemental material, sj-docx-3-dhj-10.1177_20552076261428387 for A systematic review on wearable-enabled remote health monitoring by Rita Ribeiro, Rafael Martins, Hugo Pereira, Vítor Crista, Júlio Souza, Rute Almeida, Diogo Martinho, Luís Conceição, Alberto Freitas and Goreti Marreiros in DIGITAL HEALTH

sj-pdf-4-dhj-10.1177_20552076261428387 - Supplemental material for A systematic review on wearable-enabled remote health monitoring Supplemental material, sj-pdf-4-dhj-10.1177_20552076261428387 for A systematic review on wearable-enabled remote health monitoring by Rita Ribeiro, Rafael Martins, Hugo Pereira, Vítor Crista, Júlio Souza, Rute Almeida, Diogo Martinho, Luís Conceição, Alberto Freitas and Goreti Marreiros in DIGITAL HEALTH
