# Supplementary material for: A systematic review on wearable-enabled remote health monitoring
Source: Digit Health. 2026 Feb 27;12:20552076261428387. doi: 10.1177/20552076261428387 (PMC12954012; doi:10.1177/20552076261428387)
Supplement: sj-docx-3-dhj-10.1177_20552076261428387 - Supplemental material for A systematic review on wearable-enabled remote health monitoring [file sj-docx-3-dhj-10.1177_20552076261428387.docx]

**Supplementary Material**

**Final studies included**

| Author | Title |
| --- | --- |
| Adams et al., (2021) | Adaptive Goals and Reinforcement Timing to Increase Physical Activity in Adults: A Factorial Randomized Trial |
| Althobiani et al., (2023) | Evaluating a Remote Monitoring Program for Respiratory Diseases: Prospective Observational Study |
| Anand et al., (2021) | Group-Based Exercise in CKD Stage 3b to 4: A Randomized Clinical Trial |
| Batsis et al., (2021) | Feasibility and acceptability of a technology-based, rural weight management intervention in older adults with obesity |
| Bell et al., (2020) | A Portable System for Remote Rehabilitation Following a Total Knee Replacement: A Pilot Randomized Controlled Clinical Study |
| Blair et al., (2021) | A Home-Based Mobile Health Intervention to Replace Sedentary Time With Light Physical Activity in Older Cancer Survivors: Randomized Controlled Pilot Trial |
| Bonometti et al., (2023) | Usability of a continuous oxygen saturation device for home telemonitoring |
| Breteler et al., (2020) | Wireless Remote Home Monitoring of Vital Signs in Patients Discharged Early After Esophagectomy: Observational Feasibility Study |
| Chae et al., (2020) | Development and Clinical Evaluation of a Web-Based Upper Limb Home Rehabilitation System Using a Smartwatch and Machine Learning Model for Chronic Stroke Survivors: Prospective Comparative Study |
| Charkviani et al., (2023) | Conceptualization of Remote Patient Monitoring Program for Patients with Complex Medical Illness on Hospital Dismissal |
| Chen et al., (2020) | Wearable Motion Sensor Device to Facilitate Rehabilitation in Patients With Shoulder Adhesive Capsulitis: Pilot Study to Assess Feasibility |
| Cochen De Cock et al., (2021) | BeatWalk: Personalized Music-Based Gait Rehabilitation in Parkinson's Disease |
| Davies et al., (2021) | Demonstrating the feasibility of digital health to support pediatric patients in South Africa |
| Ding et al., (2021) | Design, deployment, and usability of a mobile system for cardiovascular health monitoring within the electronic Framingham Heart Study |
| Downing et al., (2023) | Results of a postoperative telemedicine trial after cardiac surgery and incorporation into practice |
| Duarte-Rojo et al., (2023) | Use of a Mobile-Assisted Telehealth Regimen to Increase Exercise in Transplant Candidates: A Home-Based Prehabilitation Pilot and Feasibility Trial |
| Fanning et al., (2020) | A Mobile Health Behavior Intervention to Reduce Pain and Improve Health in Older Adults With Obesity and Chronic Pain: The MORPH Pilot Trial |
| Gaßner et al., (2022) | The Effects of an Individualized Smartphone-Based Exercise Program on Self-defined Motor Tasks in Parkinson Disease: Pilot Interventional Study |
| Ha et al., (2022) | A Digital Educational Intervention With Wearable Activity Trackers to Support Health Behaviors Among Childhood Cancer Survivors: Pilot Feasibility and Acceptability Study |
| Harzand et al., (2023) | Effects of a patient-centered digital health intervention in patients referred to cardiac rehabilitation: the Smart HEART clinical trial |
| Hesketh et al., (2025) | Mobile Health Biometrics to Enhance Exercise and Physical Activity Adherence in Type 2 Diabetes (MOTIVATE-T2D): a decentralised feasibility randomised controlled trial delivered across the UK and Canada |
| Indraratna et al., (2021) | Trials and Tribulations: mHealth Clinical Trials in the COVID-19 Pandemic |
| Khusial et al., (2020) | Effectiveness of myAirCoach: A mHealth Self-Management System in Asthma |
| Kikuchi et al., (2021) | Feasibility of home-based cardiac rehabilitation using an integrated telerehabilitation platform in elderly patients with heart failure: A pilot study |
| Klein et al., (2024) | Lessons learned from a multimodal sensor-based eHealth approach for treating pediatric obsessive-compulsive disorder |
| LeBaron et al., (2023) | Describing and visualizing the patient and caregiver experience of cancer pain in the home context using ecological momentary assessments |
| Leenen et al., (2023) | Usability of a digital health platform to support home hospitalization in heart failure patients: a multicentre feasibility study among healthcare professionals |
| Leitner et al., (2022) | An mHealth Lifestyle Intervention Service for Improving Blood Pressure using Machine Learning and IoMTs |
| Leitner et al., (2024) | The Effect of an AI-Based, Autonomous, Digital Health Intervention Using Precise Lifestyle Guidance on Blood Pressure in Adults With Hypertension: Single-Arm Nonrandomized Trial |
| Li et al., (2024) | Digital health interventions to promote healthy lifestyle in hemodialysis patients: an interventional pilot study |
| Li et al., (2025) | Digital Therapeutics–Based Cardio-Oncology Rehabilitation for Lung Cancer Survivors: Randomized Controlled Trial |
| Lin et al., (2024) | Evaluation of a Telemonitoring System Using Electronic National Early Warning Scores for Patients Receiving Medical Home Care: Pilot Implementation Study |
| Liu et al., (2022) | Wearable Smartwatch Facilitated Remote Health Management for Patients Undergoing Transcatheter Aortic Valve Replacement |
| Loh et al., (2022) | A single-arm pilot study of a mobile health exercise intervention (GO-EXCAP) in older patients with myeloid neoplasms |
| Matthews et al., (2024) | Cloud-Integrated Smart Nanomembrane Wearables for Remote Wireless Continuous Health Monitoring of Postpartum Women |
| Mehta et al., (2020) | Effect of Remote Monitoring on Discharge to Home, Return to Activity, and Rehospitalization After Hip and Knee Arthroplasty: A Randomized Clinical Trial |
| Moore et al., (2025) | Using technology for patient-centered care at home after CAR T-cell therapy or stem cell transplant: a prospective feasibility study |
| Motolese et al., (2023) | Feasibility and efficacy of an at-home, smart-device aided mindfulness program in people with Multiple Sclerosis |
| O'Connor et al., (2025) | Healthy at Home for COPD: An Integrated Digital Monitoring, Treatment, and Pulmonary Rehabilitation Intervention |
| Oftring et al., (2025) | Feasibility, utility, usability and acceptance of a multimodal telemonitoring for COVID-19 patients in general practitioners practices in Germany: a mixed methods study with patients |
| Patel et al., (2022) | Building a Real-Time Remote Patient Monitoring Patient Safety Program for COVID-19 Patients |
| Purnell et al., (2023) | Acceptability and Usability of a Wearable Device for Sleep Health Among English- and Spanish-Speaking Patients in a Safety Net Clinic: Qualitative Analysis |
| Sañudo et al., (2024) | A randomized controlled mHealth trial that evaluates social comparison-oriented gamification to improve physical activity, sleep quantity, and quality of life in young adults |
| Stubberud et al., (2020) | Biofeedback Treatment App for Pediatric Migraine: Development and Usability Study |
| Sze et al., (2023) | StepAdd: A personalized mHealth intervention based on social cognitive theory to increase physical activity among type 2 diabetes patients |
| Toh et al., (2023) | Usability of a wearable device for home-based upper limb telerehabilitation in persons with stroke: A mixed-methods study |
| Toh et al., (2025) | ‘Smart reminder’: A feasibility pilot study on the effects of a wearable device treatment on the hemiplegic upper limb in persons with stroke |
| Tzeng et al., (2025) | Effect of a Digitally Monitored Walking Program on Functional and Psychological Outcomes in Individuals with Mild Traumatic Brain Injury: A Randomized Controlled Trial |
| van den Bergh et al., (2023) | Usability and utility of a remote monitoring system to support physiotherapy for people with Parkinson's disease |
| van Ede et al., (2022) | Continuous remote monitoring in post-bariatric surgery patients: development of an early warning protocol |
| Walter et al., (2023) | Financial and Clinical Impact of Virtual Care During the COVID-19 Pandemic: Difference-in-Differences Analysis |
| Wilmink et al., (2020) | Artificial Intelligence-Powered Digital Health Platform and Wearable Devices Improve Outcomes for Older Adults in Assisted Living Communities: Pilot Intervention Study |
| Wong et al., (2022) | Daily ambulatory remote monitoring system for drug escalation in chronic heart failure with reduced ejection fraction: pilot phase of DAVID-HF study |
| Wu et al., (2024) | Feasibility of a wearable self-management application for patients with COPD at home: a pilot study |
| Wurzer et al., (2021) | Remote monitoring of COVID-19 positive high-risk patients in domestic isolation: A feasibility study |
