# Supplementary material for: A systematic review on wearable-enabled remote health monitoring
Source: Digit Health. 2026 Feb 27;12:20552076261428387. doi: 10.1177/20552076261428387 (PMC12954012; doi:10.1177/20552076261428387)
Supplement: sj-pdf-4-dhj-10.1177_20552076261428387 - Supplemental material for A systematic review on wearable-enabled remote health monitoring [file sj-pdf-4-dhj-10.1177_20552076261428387.pdf]

NIHR | National Institute for Health and Care Research

PROSPERO
International prospective register of systematic reviews

A Systematic Review on Wearable-enabled Remote Health Monitoring

Rita Ribeiro, Rafael Gonçalves Martins, Hugo Pereira, Vítor Crista, Julio Souza, Rute Almeida, Diogo Martinho, Luís Conceição, Alberto Freitas, Goreti Marreiros

Citation
Rita Ribeiro, Rafael Gonçalves Martins, Hugo Pereira, Vítor Crista, Julio Souza, Rute Almeida, Diogo Martinho, Luís Conceição, Alberto Freitas, Goreti Marreiros. A Systematic Review on Wearable-enabled Remote Health Monitoring. PROSPERO 2025 CRD420251156256. Available from https://www.crd.york.ac.uk/PROSPERO/view/CRD420251156256.

REVIEW TITLE AND BASIC DETAILS

Review title
A Systematic Review on Wearable-enabled Remote Health Monitoring

Condition or domain being studied
Population health; Remote digital monitoring ; Digital health; Telehealth; Artificial intelligence; Patient engagement; Patient monitoring

The review addresses the integration of sensor-based and mobile technologies into remote healthcare delivery. It synthesizes evidence on their methodological rigor, data types, and evaluation outcomes related to effectiveness, patient engagement, and usability in domiciliary health monitoring.

Rationale for the review
Remote health monitoring is expanding rapidly, yet evidence about its effectiveness and real-world implementation in home settings remains fragmented. Primary studies vary widely in design, duration, populations, device types and monitoring strategies, and often lack long-term follow-up or appropriate comparators. Reporting on feedback mechanisms is heterogeneous and rarely grounded in explicit behavioural theory, while most studies still rely on traditional statistics with limited integration of machine learning. Prior reviews are typically

disease- or device-specific, leaving a cross-condition synthesis of methods, outcomes and implementation gaps underexplored.

This review consolidates evidence from recent, real-world studies of wearable device-based monitoring at home to map technologies used, evaluation metrics, and patient/clinician-facing features, and to assess effects on engagement, usability and health outcomes. By identifying consistent benefits and recurring limitations (e.g., small samples, short durations, scarce clinician alerts, limited AI use), it aims to inform future trial design, outcome standardisation, and the development of scalable, patient-centred remote care.

Review objectives

Main objective: "What is the current state-of-the-art regarding the use of wearable technologies for remote health monitoring?"

Secondary research questions:

SRQ1 - What are the main wearables technologies employed in the field of remote health monitoring?

SRQ2 - What are the health domains most covered by currently available wearable technologies?

SRQ3 - What are the most frequent feedback strategies/mechanisms considered in the context of remote health monitoring?

SRQ4 - Which artificial intelligence methods are most used in the context of remote health monitoring?

Keywords

Wearable technology; Remote monitoring; Artificial intelligence; Patient engagement; Health Data Analytics

Country

Portugal

ELIGIBILITY CRITERIA

Population

Included

Studies including human participants of any age, sex, or health condition monitored remotely using wearable or mobile digital devices in domiciliary or home-based healthcare contexts.

Eligible populations include individuals with chronic, cardiovascular, respiratory, neurological, oncological, or metabolic diseases, as well as participants involved in rehabilitation, prevention, wellbeing, or transitional care programs.

Only studies involving real users (not simulations) and reporting physiological or behavioural

health data were included.

Excluded
Studies not involving human participants or conducted in hospital-only or laboratory settings.

Studies using simulated, animal, or synthetic data were excluded, as well as those not applying wearable monitoring technologies.

Papers lacking detailed participant information, or studies focusing exclusively on technical development without user evaluation, were also excluded.

Intervention(s) or exposure(s)

Included
Digital health intervention; Remote digital monitoring ; mHealth; Sensor Device

Studies evaluating wearable device-based interventions for remote health monitoring, including the use of sensors to collect physiological or behavioural data.

Eligible interventions aim to monitor, support, or improve health outcomes through continuous or periodic tracking in home or domiciliary settings.

Both commercial and research-developed devices were included when applied in real user contexts including home or domiciliary settings.

Excluded
Studies not involving a wearable monitoring component, or those focusing solely on technical development without user deployment.

Interventions conducted exclusively in hospital, laboratory, or simulation environments were excluded, as well as studies that used digital questionnaires or teleconsultations without physiological or behavioural data collection.

Publications not reporting an implemented intervention or lacking outcome evaluation were also excluded.

Comparator(s) or control(s)

Included
PICO tags selected: Usual Care

Studies that compared wearable monitoring interventions with standard or usual care, no intervention, or non-digital monitoring approaches were included.

Comparators could consist of routine clinical follow-up, traditional paper-based tracking, or conventional home monitoring without feedback or automation.

Excluded
Comparisons based solely on simulation, algorithmic benchmarking, or laboratory testing without real participants were not considered valid comparators.

Study design

Both randomized and nonrandomized study types will be included.

Included
Randomised controlled trials, non-randomised intervention studies, observational studies, feasibility and pilot studies, mixed-methods studies, and qualitative studies will be included.

All studies must involve human participants monitored remotely through wearable technologies (e.g., wristbands, smartwatches, patch sensors, rings, or similar devices) in domiciliary healthcare contexts.

Comparative and non-comparative designs are eligible if they evaluate clinical, behavioural, or usability outcomes related to wearable-based remote monitoring.

Excluded
Studies not involving wearable devices for health monitoring, including those using only teleconsultations or non-wearable sensors will be excluded.

Other exclusions include studies without human participants, simulation-based research, reviews, protocols, technical papers without user testing, or publications lacking primary data on wearable use.

Context
This review focuses on studies conducted in domiciliary, home-based, or community healthcare settings, where participants used wearable devices to monitor health parameters remotely.

Eligible studies include interventions or observational research performed outside hospital or clinical environments, involving real-world use of wearable technologies for continuous or periodic health tracking.

Hospital-only or simulation-based studies were excluded, as the review aims to capture the applicability and effectiveness of wearables in real-life remote healthcare contexts including home or domiciliary settings.

TIMELINE OF THE REVIEW

Date of first submission to PROSPERO
30 October 2025

Review timeline
Start date: 25 September 2025. End date: 30 November 2025.

Date of registration in PROSPERO
03 November 2025

AVAILABILITY OF FULL PROTOCOL

Availability of full protocol

A full protocol has been written but is not available because:

The full protocol is not being made publicly available at this time because the review is being submitted for presentation at an academic conference. To maintain the integrity of the peer review process and to comply with submission guidelines.

SEARCHING AND SCREENING

Search for unpublished studies

Only published studies will be sought.

Main bibliographic databases that will be searched

The main databases to be searched are *PubMed* and *SCI - Science Citation Index*.

Other important or specialist databases that will be searched

In addition to the main health and biomedical sources (PubMed and Web of Science), IEEE Xplore and ScienceDirect were searched to capture studies published in engineering, computer science, and interdisciplinary journals relevant to wearable-based remote health monitoring.

Search language restrictions

The review will only include studies published in English.

Search date restrictions

Databases will be searched for articles published from 1 January 2020, there are no search end date restrictions.

Other methods of identifying studies

No other methods will be used.

Link to search strategy

A full search strategy has been uploaded to PROSPERO. The PDF may be accessed through this link https://www.crd.york.ac.uk/PROSPEROFILES/5a71494a4777e86cb95d63a2f9244ba1.pdf.

Selection process

Studies will be screened independently by at least two people (or person/machine combination) with a process to resolve differences.

Other relevant information about searching and screening

When a conflict is identified between two reviewers a 3rd reviewer will also evaluate the conflicted work in order to reach a majority decision.

DATA COLLECTION PROCESS

Data extraction from published articles and reports

Data will be extracted independently by at least two people (or person/machine combination) with a process to resolve differences.

Authors will be asked to provide any required data not available in published reports.

Study risk of bias or quality assessment

Risk of bias will be assessed using: *Cochrane RoB-2* and *ROBINS-I*

Data will be assessed independently by at least two people (or person/machine combination) with a process to resolve differences.

Additional information will **not** be sought from study investigators if required information is unclear or unavailable in the study publications/reports.

Reporting bias assessment

Direct methods for assessing the risk of bias due to missing results include comparing outcomes and analyses pre-specified in study registers, protocols, and statistical analysis plans with results that were available in study reports.

Certainty assessment

Methods considered include common factors such as precision of the effect estimate (or sample size), consistency of findings across studies, study design limitations and missing results (risk of bias).

OUTCOMES TO BE ANALYSED

Main outcomes

The main outcomes include measures of effectiveness, usability, and patient engagement in wearable-based remote health monitoring.

Clinical or physiological outcomes comprise improvements in disease management, symptom control, physical activity, sleep quality, heart rate or vital-sign stability, and prevention indicators.

Behavioural and process-related outcomes include adherence to the intervention, system usability (SUS or equivalent scales), user satisfaction, and frequency of use or data transmission.

Studies reporting feedback mechanisms between user and clinician, or AI-based monitoring performance (e.g., accuracy, sensitivity, specificity), were also included as part of the primary outcomes assessed.

Additional outcomes

Additional outcomes will be included as necessary, based on the scope and characteristics of the studies identified during the review process.

PLANNED DATA SYNTHESIS

Strategy for data synthesis

Data will be synthesized through a structured, multi-phase approach. A narrative synthesis will first be conducted to summarize the main study characteristics, including population, health condition, study design, type of wearable device (e.g., smartwatch, wristband, sensor patch), monitoring setting (home-based or community healthcare), and intervention purpose (e.g., physical activity tracking, cardiovascular monitoring, sleep assessment).

Outcomes will be grouped into clinical/physiological, behavioural, usability, and technical performance domains. Quantitative data will be summarized descriptively, when available.

If sufficient homogeneity exists among comparable studies, a quantitative synthesis (meta-analysis) may be considered for specific outcomes (e.g., accuracy of wearable-derived measurements versus reference standards). Heterogeneity will be assessed using the I^2 statistic and subgroup analyses may be performed based on device type, signal modality, or study population.

All synthesis procedures will adhere to PRISMA guidelines and the methodological recommendations of the Cochrane Handbook for Systematic Reviews of Interventions.

Studies will also be evaluated regarding risk of bias (RoB 2 and ROBIN-I), according to the type of study (RCT or NRSI).

CURRENT REVIEW STAGE

Stage of the review at this submission 1 change

Review stage	Started	Completed
Pilot work	✓	✓
Formal searching/study identification	✓	✓
Screening search results against inclusion criteria	✓	✓
Data extraction or receipt of IPD	✓	✓
Risk of bias/quality assessment	✓	✓
Data synthesis	✓	✓

Review status

The review is completed.

Publication of review results

Results of the review will be published in English.

REVIEW AFFILIATION, FUNDING AND PEER REVIEW

Review team members

Rita Ribeiro (review guarantor and contact) ORCID: 0009-0005-8882-6214. ISEP / GECAD. Portugal.

No conflict of interest declared.

Rafael Gonçalves Martins. ORCID: 0000-0003-1222-8136. ISEP / GECAD. Portugal.

No conflict of interest declared.

Hugo Pereira. ORCID: 0009-0002-2939-0762. ISEP / GECAD. Portugal.

No conflict of interest declared.

Vítor Crista. ORCID: 0000-0002-8794-6354. ISEP / GECAD. Puerto Rico.

No conflict of interest declared.

Julio Souza. ORCID: 0000-0002-8576-1903. ISEP / GECAD. Portugal.

No conflict of interest declared.

Rute Almeida. ORCID: 0000-0001-7755-5002. FMUP / RISE-Health, MEDCIDS. Portugal.

No conflict of interest declared.

Diogo Martinho. ORCID: 0000-0003-1683-4950. ISEP / GECAD. Portugal.

No conflict of interest declared.

Luís Conceição. ORCID: 0000-0003-3454-4615. ISEP / GECAD. Portugal.

No conflict of interest declared.

Alberto Freitas. ORCID: 0000-0003-2113-9653. FMUP / RISE-Health, MEDCIDS. Portugal.

No conflict of interest declared.

Goreti Marreiros. ORCID: 0000-0003-4417-8401. ISEP / GECAD. Portugal.

No conflict of interest declared.

Named contact

Rita Ribeiro (rtapr@isep.ipp.pt). ORCID: 0009-0005-8882-6214. ISEP / GECAD. Portugal.

Review affiliation

GECAD, Institute of Engineering, Polytechnic of Porto

RISE-Health, Information and Health, Decision Sciences, Faculty of Medicine, University of Porto

Funding source

Grant number
See additional details

Additional non-commercial funding information
European Regional Development Fund (ERDF)

Portuguese Foundation for Science and Technology (FCT)

Additional information about funding
Funded by the European Regional Development Fund (ERDF), project number COMPETE2030-FEDER-00391100, and funded by National Funds through the Portuguese Foundation for Science and Technology (FCT) under the R&D Units Project Scope, UIDB/00760/2020 (https://doi.org/10.54499/UIDB/00760/2020). RA is supported by FCT under the grant agreement CEECINST/00056/2021/CP2804/CT0004.

Peer review
Review is being done following Cochrane method, with peer review being part of the review production process.

ADDITIONAL INFORMATION

Review conflict of interest
Declared individual interests are recorded under team member details.. No additional interests are recorded for this review.

Medical Subject Headings
Artificial Intelligence; Feedback; Humans; Patient Participation; Wearable Electronic Devices

Revision note 1 change
Update on the work status.

SIMILAR REVIEWS

Check for similar records already in PROSPERO
PROSPERO identified a number of existing PROSPERO records that were similar to this one (last check made on 30 October 2025). These are shown below along with the reasons given by that the review team for the reviews being different and/or proceeding.

- The impact of remote monitoring technologies on health outcomes and quality of life in patients with atrial fibrillation: A systematic review. [published 2 June 2025] [CRD420251009323]. The review was judged **not to be similar**
- Economic Evaluations of Digital Health Technologies: A Systematic Review of Methodological Approaches and Outcome Assessments [published 23 November 2024] [CRD42024613511]. The review was judged **not to be similar**
- Equipment and Data Collection in Wearable Remote Patient Monitoring in COPD – A Systematic Review [published 12 April 2023] [CRD42023411492]. The review was judged **not to be similar**

- Effect of AI/ML in diagnostic assistance (healthcare expert) and patient monitoring remotely (using wearabledevices) [published 28 April 2023] [CRD42023418012]. The review was judged **not to be similar**
- Remote Monitoring in Heart Failure: A Systematic Review and Meta-analysis of Randomized Trials [published 1 October 2025] [CRD420251109400]. The review was judged **not to be similar**
- Systematic Review of Wearable Technology and Artificial Intelligence for Toxicity Management in Breast Cancer Treatment: Applications, Outcomes, and Future Directions [published 12 February 2025] [CRD42025645803]. The review was judged **not to be similar**

PROSPERO version history 1 change

- Version 1.4, published 30 Nov 2025
- Version 1.3, published 27 Nov 2025
- Version 1.2, published 18 Nov 2025
- Version 1.1, published 13 Nov 2025
- Version 1.0, published 03 Nov 2025

Disclaimer

The content of this record displays the information provided by the review team. PROSPERO does not peer review registration records or endorse their content.

PROSPERO accepts and posts the information provided in good faith; responsibility for record content rests with the review team. The guarantor for this record has affirmed that the information provided is truthful and that they understand that deliberate provision of inaccurate information may be construed as scientific misconduct.

PROSPERO does not accept any liability for the content provided in this record or for its use. Readers use the information provided in this record at their own risk.

Any enquiries about the record should be referred to the named review contact
